# A Two-Stage Coarse-to-Fine Framework for Sparse Crowd Density Prediction in Digital Twin-Based Safety Monitoring

**DOI:** 10.3390/s26134094

**Published:** 2026-06-27

**Authors:** Younghwan Jeong, SoHyeon Kim, Jinyoung Lee, Donghoon Lee, Taemin Hwang, Won Gi Choi

**Affiliations:** 1 Korea Electronics Technology Institute, Seongnam-si 13509, Gyeonggi-do, Republic of Korea; cjstntjd@keti.re.kr (Y.J.); shk980903@keti.re.kr (S.K.); jylee@keti.re.kr (J.L.); dh_lee99@keti.re.kr (D.L.); 2 Sonatus Korea, 660, Daewangpangyo-ro, Bundang-gu, Seongnam-si 13494, Gyeonggi-do, Republic of Korea; taemin.hwang@sonatus.com

**Keywords:** digital twin, crowd density prediction, spatio-temporal sparsity, crowd safety monitoring

## Abstract

Crowd-related disasters in dense public spaces unfold into hazardous situations within seconds, repeatedly demonstrating that reactive response alone is insufficient to minimize damage. This reality has intensified the need for monitoring systems that can proactively forecast congestion before it reaches a critical level. Digital twin platforms address this need by providing an operational substrate that represents crowd states on a unified bird’s-eye-view (BEV) grid, on which a predictive module can forecast where congestion will emerge. However, conventional AI-based single-stage dense prediction models are intrinsically ill-suited to this role: although crowd congestion is sparse in both space and time, these models apply uniform high-resolution computation across the entire BEV domain, wasting computation and biasing optimization toward dominant background regions. In this paper, we propose a two-stage coarse-to-fine framework that operates as the predictive module of the digital twin and explicitly exploits the spatio-temporal sparsity of crowd congestion. The first stage, CoarseSTFormer, performs efficient global screening on a low-resolution BEV input to coarsely identify a set of density-critical candidate regions. The second stage, SparseQueryDecoder, selectively reconstructs high-resolution responses only on the identified candidates, rather than uniformly upsampling the entire BEV grid. In simulation environments with up to 20,000 pedestrian agents, the proposed framework matches the strongest dense baseline in reconstruction quality while delivering the most balanced variance profile across grid scales. At inference, it further reduces GPU energy consumption by 1.9× to 5.0× and computational cost (FLOPs) by 3.8× to 54×, demonstrating its practicality as a resource-efficient predictive module that satisfies both accuracy and efficiency.

## 1. Introduction

Crowd-related disasters in dense public spaces share a common operational feature: they unfold within seconds and leave little margin for reactive intervention [1,2,3]. From transit hubs to outdoor festivals, localized congestion can escalate into compressive crowd pressure faster than human operators can respond. The decisive factor in mitigating such hazards is therefore not how quickly the present state is observed, but how reliably the future state can be anticipated.

Digital twin platforms [4,5,6] have emerged as a natural substrate for this anticipatory monitoring. By representing crowd states on a unified bird’s-eye-view (BEV) grid [7,8,9], such a twin provides a single spatial frame on which prediction, visualization, and operator decision-making can in principle be co-located. The component that converts this substrate into actionable foresight is the predictive module: it must indicate, on the BEV grid, where and when density-critical regions are likely to emerge.

Yet existing prediction architectures [10,11,12,13], when placed in this role, exhibit a structural mismatch with the phenomenon they are asked to forecast. Crowd congestion is sparse in both space and time, since severe density events occupy only a small fraction of the BEV grid and arise in limited intervals, but conventional single-stage dense models apply uniform high-resolution computation across the entire grid. This uniform allocation has two compounding consequences. First, inference cost scales with the full BEV resolution, which is difficult to sustain at the latencies required for real-time monitoring. Second, the loss signal is dominated by abundant background pixels, biasing the model toward reproducing non-critical regions instead of localizing the rare events that matter for safety. As a result, dense predictors tend to be simultaneously expensive and unreliable in precisely the regions where reliability is most needed.

We argue that resolving this mismatch requires aligning the computational structure of the predictor with the statistical structure of the data. To this end, we propose a two-stage coarse-to-fine framework that serves as the predictive module of the digital twin. The first stage performs efficient global screening on a low-resolution BEV input to identify a compact set of density-critical candidate regions; the second stage reconstructs high-resolution responses only on those candidates, rather than refining the entire BEV grid. By decoupling global screening from local refinement, the framework concentrates both computation and supervision on safety-relevant regions, addressing the two failure modes of dense prediction at once.

The main contributions of this paper are summarized as follows:

- We identify a structural mismatch between dense prediction architectures and the spatio-temporal sparsity of crowd congestion, and frame it as the central design constraint for the predictive module of a digital twin-based monitoring system.
- We propose a two-stage coarse-to-fine framework, consisting of **CoarseSTFormer** for low-resolution global screening and **SparseQueryDecoder** for sparse high-resolution refinement, that aligns computation with the distribution of safety-relevant information on the BEV grid.
- We empirically validate the framework in large-scale pedestrian simulations, showing that it preserves the accuracy of dense baselines while substantially reducing inference cost and remaining robust across heterogeneous grid configurations.

The remainder of this paper is organized as follows: Section 2 reviews related work on crowd density estimation, video-based spatio-temporal prediction, and digital twin-based monitoring. Section 3 outlines the digital twin context in which the proposed predictive module operates. Section 4 details the proposed two-stage coarse-to-fine framework, including its global screening and sparse refinement components. Section 5 presents the simulation setup and experimental results, covering accuracy, robustness, and inference cost. Section 6 concludes the paper and outlines directions for future work.

## 2. Related Works

### 2.1. Digital Twin-Based Crowd Management

A digital twin is generally understood as a virtual representation of a physical system that is continuously updated with real-world data and used for monitoring, simulation, and decision support [5,6,14,15]. Unlike static simulators, digital twins emphasize the dynamic linkage between physical and virtual environments, which makes them particularly suitable for safety-critical applications that require real-time situational awareness.

Building on this paradigm, several studies have applied digital twin technology to crowd management in public spaces. Villanueva et al. [8] proposed a smart-city platform for large-scale events in which IoT, camera, and social-media streams are integrated into a 3D environment to support situational awareness. Simulation-based digital twins have been used in transportation facilities to forecast pedestrian flow and assist evacuation planning [9], and Casadei et al. [16] extended the concept to crowd-aware services by modeling the collective behavior of human groups.

Despite this progress, two limitations recur across existing digital twin studies on crowd management. First, most work targets structured, infrastructure-rich environments such as smart cities and railway stations, where spatial layouts and sensing coverage are stable; the use of digital twins as a predictive substrate for less structured deployments has received considerably less attention. Deploying such crowd-facing monitoring systems at scale also raises user-centric considerations, including privacy and the burden placed on sensed individuals, which have been systematically examined in the crowd-powered positioning literature [17]. Second, even when prediction is included, many systems remain oriented toward current-state visualization rather than anticipatory forecasting of where and when congestion will emerge. Yet the value of a digital twin for crowd safety hinges precisely on the latter: without a predictive module that produces actionable foresight over the unified spatial frame, the twin reduces to a visualization layer. This observation motivates our view of the digital twin as an operational substrate whose effectiveness is ultimately determined by its predictive module, which is the focus of this work.

### 2.2. AI-Based Crowd Prediction

Camera-based crowd analysis has been extensively studied for density estimation, anomaly detection, and tracking. CNN-based density estimators recover per-frame density maps in congested scenes [2,3,18,19], while anomaly-detection methods identify irregular motion patterns from spatio-temporal cues [20,21,22]. These approaches, however, are confined to interpreting the current state and do not forecast how congestion will evolve, which is the predictive requirement of a digital twin module.

Pedestrian-level trajectory forecasting addresses the temporal dimension. Social-LSTM [23], Social-GAN [24], and Social-STGCNN [25] predict future trajectories by modeling pairwise interactions among individuals. While effective in structured scenes, they rely on per-pedestrian tracking and operate at the individual level rather than producing the environment-scale density forecast that operators of a digital twin actually need.

More recently, general spatio-temporal predictors have been applied to future-frame forecasting. SimVP [10] provides a lightweight CNN-based baseline; ViT [11] and Swin Transformer [12] model global and hierarchical dependencies via self-attention; and Mamba-based state-space models [13] offer linear-time long-range sequence modeling. These architectures broaden the design space for environment-level prediction, but when applied as the predictive module of a digital twin they share a common structural limitation: they perform uniform dense computation over the entire high-resolution BEV grid. Because severe congestion is sparse in both space and time, this uniform allocation has two consequences. The bulk of computation is spent on non-critical background regions, which makes real-time deployment on the full BEV resolution difficult, and the training loss is dominated by easy background pixels, biasing optimization away from the rare regions where safety risks actually emerge. As a result, these models tend to be simultaneously expensive and unreliable in precisely the regions where reliability matters most.

To our knowledge, no prior crowd-prediction architecture is explicitly designed around the spatio-temporal sparsity of congestion events, as summarized in Table 1. Our work fills this gap by aligning the computational structure of the predictor with the statistical structure of the data: a coarse global screening stage identifies density-critical candidate regions on a low-resolution BEV, and a sparse high-resolution decoder reconstructs fine-grained responses only on those candidates. This positions the proposed framework not as another general-purpose predictor, but as a sparsity-aware predictive module purpose-built for digital twin-based crowd safety monitoring.

## 3. System Model

This section outlines the digital twin-based crowd safety management system that forms the operating context of the proposed prediction module. The system converts raw observations of a public space into actionable decision support through four functional stages, and the predictive analysis stage among them is the subject of this paper. The remaining stages are described at the level of their roles and interfaces, through which the prediction module interacts with the rest of the system.

### 3.1. Overall Architecture

**Figure 1:**
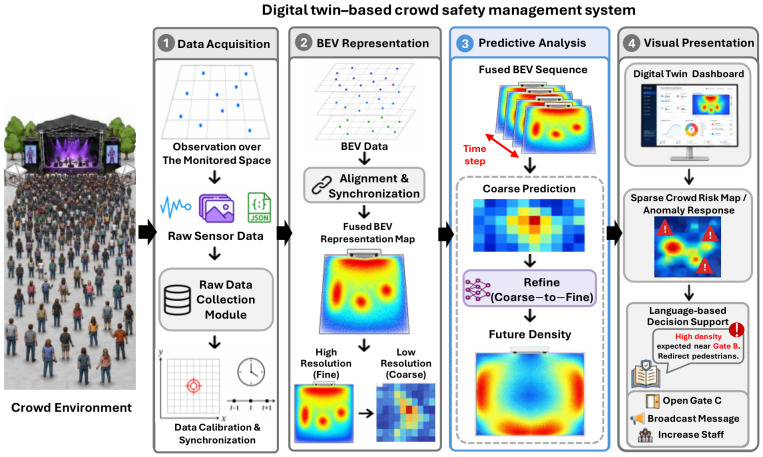
Conceptual overview of the digital twin-based crowd safety management system in which the proposed prediction module operates. The predictive analysis stage (third panel) is the contribution of this paper; the surrounding stages provide its operating context. The prediction module connects to that context only through a BEV input sequence and a density-critical output map.

The system is organized as a four-stage pipeline that reflects the operational logic of crowd safety management: *observe*, *represent*, *predict*, and *communicate*. The four stages, illustrated conceptually in Figure 1, are summarized as follows:

1. **Data Acquisition:** Distributed on-site sensing devices across the monitored space acquire evidence of pedestrian activity in real time, which the subsequent stage organizes into the representation consumed by the prediction module.
2. **BEV Representation:** The acquired evidence is consolidated into a unified spatio-temporal representation aligned on a bird’s-eye-view (BEV) reference frame.
3. **Predictive Analysis:** The proposed two-stage coarse-to-fine framework, detailed in Section 4, operates on the BEV representation to forecast density-critical regions over a near-future horizon.
4. **Visual Presentation:** The forecast outcomes are delivered to human operators through an interpretable visual interface, complemented by a language-based decision-support layer.

The stages communicate through well-defined interfaces so that each can be developed, replaced, or upgraded independently. The prediction module in particular interacts with the rest of the system only through its input, a sequence of BEV representations, and its output, a density-critical response map; the sensing, calibration, and operator-facing infrastructure behind the other stages belongs to a separate engineering layer.

### 3.2. Input Context: From On-Site Observation to the BEV Representation

The first two stages define the input of the prediction module, which is coupled to the rest of the system through a single interface. The monitored space is observed by distributed sensing devices, and the registration stage consolidates these observations onto a common BEV reference frame over the ground plane, aligned on a shared discrete-time grid. Because the module consumes only the resulting representation, it is agnostic to the sensing and fusion choices upstream: any configuration that yields a consistent BEV sequence presents the same interface. That representation is a temporally ordered sequence of BEV frames, each summarizing the pedestrian distribution at a given time step, and it constitutes the sole input assumed by the proposed framework. The experiments in Section 5 instantiate this interface with large-scale pedestrian simulations.

### 3.3. Predictive Analysis

The predictive analysis stage transforms an observed BEV sequence into an estimate of where and when crowd congestion is likely to emerge over a near-future horizon, expressed on the same BEV reference frame. As discussed in Section 1, the principal challenge here is the structural mismatch between conventional dense predictors and the spatio-temporal sparsity of congestion. The stage is therefore instantiated as the proposed two-stage coarse-to-fine framework, whose design and training are detailed in Section 4; at the system level, it delivers a high-resolution density-critical response map to the visual presentation stage.

### 3.4. Output Context: Visual Presentation and Decision Support

The visual presentation stage renders the predicted density-critical regions on the BEV reference frame with interpretable encodings of location, extent, and confidence so that operators can localize emerging risks without interpreting raw probability values. A complementary language-based decision-support layer translates the spatial forecast into concise situational summaries and response recommendations grounded in domain-specific safety guidelines; its design and evaluation form a separate line of work.

## 4. Proposed Method

**Figure 2:**
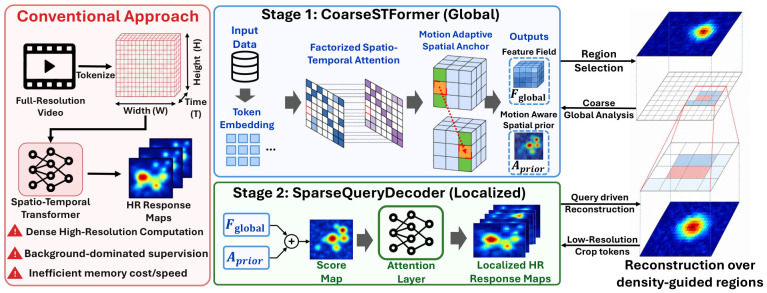
Overview of the proposed two-stage coarse-to-fine framework.

The overall pipeline of the proposed framework is illustrated in Figure 2. Crowd density prediction requires modeling the temporal dynamics of moving agents under complex and non-stationary conditions. In real-world environments, extreme crowd congestion does not occur uniformly across the entire spatio-temporal domain. Instead, it emerges sparsely within localized spatial regions and limited temporal intervals. This observation implies that crowd density signals inherently exhibit spatio-temporal sparsity.

Conventional single-resolution models [10,11,12,13] do not explicitly incorporate this sparsity into their computational design. Instead, they perform a uniform dense computation over the entire high-resolution input space. However, in practice, only a small fraction of the spatio-temporal volume contains informative congestion patterns, while the majority correspond to background or nominal states with minimal variation.

This mismatch between computational allocation and information distribution results in substantial inefficiency. Specifically, computational resources are disproportionately consumed by low-information regions, thereby limiting the model’s ability to focus on fine-grained and semantically critical congestion patterns.

More critically, this issue also affects the model training dynamics. Since the majority of pixels correspond to background regions, the loss and gradient signals are dominated by easy negative samples. As a result, the model receives relatively weak supervision for rare but important high-density regions. Consequently, the network tends to optimize toward modeling dominant background distributions rather than capturing discriminative features of sparse congestion events.

To address these limitations, we propose a two-stage coarse-to-fine framework that explicitly leverages spatio-temporal sparsity. The proposed architecture consists of: (i) a low-resolution global modeling stage, termed CoarseSTFormer, which captures long-range dependencies efficiently, and (ii) a high-resolution refinement stage, termed SparseQueryDecoder, which selectively reconstructs fine-grained details only in regions identified as active candidates.

This design enables dynamic computation by decoupling global exploration from local refinement. Instead of exhaustively processing the entire high-resolution spatio-temporal volume, the model first identifies a sparse set of candidate regions on a coarse grid and subsequently allocates high-resolution computation only to these regions. This coarse-to-fine strategy not only reduces unnecessary computation but also shifts the learning focus toward informative congestion patterns, mitigating the dominance of trivial background signals and improving representation learning for rare but critical crowd events.

### 4.1. Stage 1: CoarseSTFormer—Efficient Global Spatio-Temporal Representation

**Figure 3:**
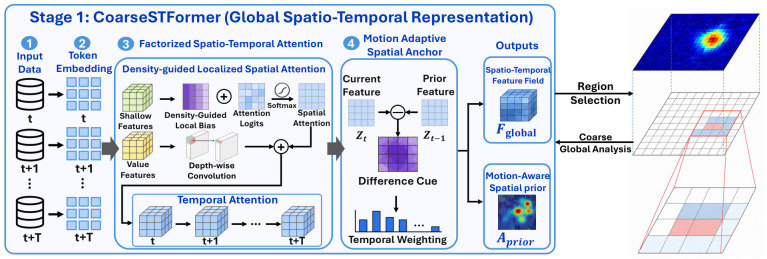
Detailed architecture of Stage 1 (CoarseSTFormer).

As outlined in the overall architecture, the objective of Stage 1, CoarseSTFormer, is to extract a coarse yet globally informative spatio-temporal representation from a low-resolution video while maintaining a tractable computational cost. This stage is designed to capture large-scale contextual dynamics before the subsequent high-resolution refinement stage focuses on locally critical regions. The detailed architecture of CoarseSTFormer is illustrated in Figure 3.

Let the low-resolution input video be

(1) $$X\in R^{B\times T\times C\times H_{lr}\times W_{lr}},$$

where *B* denotes the batch size, *T* the number of frames, *C* the number of channels, and $H_{lr},W_{lr}$ the low spatial resolution. After patch/token embedding [11], we obtain a token sequence

(2) $$Z^{\left(0\right)}\in R^{B\times T\times N\times d},$$

where $N=H_{lr}\times W_{lr}$ is the number of spatial tokens and *d* is the embedding dimension.

A naive joint spatio-temporal self-attention over all T×N tokens requires

(3) $$O\;\left({\left(TN\right)}^{2}d\right)=O\;\left(T^{2}N^{2}d\right),$$

which becomes prohibitive even for moderate *T* and *N*. Inspired by hierarchical and efficient attention designs in vision Transformers [11,12], we employ a factorized spatio-temporal attention mechanism, whose computational complexity is reduced to

(4) $$O\;\left(TN^{2}d+NT^{2}d\right).$$

This effectively reduces the quadratic complexity over the joint space to separable quadratic terms along the spatial and temporal axes.

However, a naive factorization is insufficient for crowd density estimation. Unlike generic video recognition, crowd density patterns are often highly localized in space and temporally non-stationary due to abrupt motion changes, partial occlusions, and rapidly evolving congestion regions. To address this limitation, CoarseSTFormer incorporates two structural modifications: (i) Density-guided Localized Spatial Attention, which preserves local density topology during spatial aggregation, and (ii) Motion-adaptive Spatial Anchor, which builds a temporally robust spatial prior for the next refinement stage.

#### 4.1.1. Density-Guided Localized Spatial Attention

Given the input representation at the (l−1)-th block, $Z^{\left(l-1\right)}\in R^{B\times T\times N\times d}$, we first apply layer normalization:

(5) $${\tilde{Z}}^{\left(l-1\right)}=\mathrm{LN}\;\left(Z^{\left(l-1\right)}\right).$$

For each frame, spatial self-attention is then applied over the *N* spatial tokens. To prevent standard self-attention from oversmoothing localized density peaks [3,19], we inject a density-guided local bias into the attention logits.

Specifically, shallow convolutional features $F_{\mathrm{conv}}^{\left(l\right)}\in R^{B\times T\times d_{c}\times H_{lr}\times W_{lr}}$ are first reshaped into token-level local descriptors,

(6) $$U^{\left(l\right)}=\phi \;\left(F_{\mathrm{conv}}^{\left(l\right)}\right)\in R^{B\times T\times N\times d_{b}},$$

where ϕ(·) denotes a learnable projection composed of a lightweight convolutional stem followed by a linear embedding. A pairwise local bias map is then constructed as

(7) $$M_{\mathrm{loc}}^{\left(l\right)}=\frac{\left(U^{\left(l\right)}W_{b}^{q}\right)\;{\left(U^{\left(l\right)}W_{b}^{k}\right)}^{⊤}}{\sqrt{d_{b}}}\in R^{B\times T\times N\times N},$$

where $W_{b}^{q},W_{b}^{k}\in R^{d_{b}\times d_{b}}$ are learnable projection matrices, and the resulting map is added directly to the pre-softmax spatial attention logits without further normalization, allowing the bias scale to be implicitly regulated through $W_{b}^{q}$ and $W_{b}^{k}$. The bias term encourages attention to preserve local spatial structures that are likely to correspond to density peaks or spatially coherent crowd regions.

We first compute the attention branch output as

(8) $${\hat{Z}}_{\mathrm{spa}}^{\left(l\right)}={\mathrm{MSA}}_{\mathrm{spa}}\;\left({\tilde{Z}}^{\left(l-1\right)};M_{\mathrm{loc}}^{\left(l\right)}\right)+Z^{\left(l-1\right)},$$

where

(9) $${\mathrm{MSA}}_{\mathrm{spa}}\;\left({\tilde{Z}}^{\left(l-1\right)};M_{\mathrm{loc}}^{\left(l\right)}\right)=\mathrm{Softmax}\;\left(\frac{Q_{\mathrm{spa}}^{\left(l\right)}{K_{\mathrm{spa}}^{\left(l\right)}}^{⊤}}{\sqrt{d}}+M_{\mathrm{loc}}^{\left(l\right)}\right)V_{\mathrm{spa}}^{\left(l\right)}.$$

Here, $Q_{\mathrm{spa}}^{\left(l\right)}={\tilde{Z}}^{\left(l-1\right)}W_{Q}^{\left(l\right)}$, $K_{\mathrm{spa}}^{\left(l\right)}={\tilde{Z}}^{\left(l-1\right)}W_{K}^{\left(l\right)}$, and $V_{\mathrm{spa}}^{\left(l\right)}={\tilde{Z}}^{\left(l-1\right)}W_{V}^{\left(l\right)}$ denote the projected query, key, and value tensors for spatial attention, respectively. For simplicity, we present the single-head formulation; the multi-head extension follows the standard formulation [26].

To explicitly preserve short-range spatial continuity that global attention may overlook, we further apply a depth-wise convolution branch to the value features. While the attention pathway aggregates long-range contextual dependencies across all spatial tokens, it does not explicitly impose a local neighborhood prior, which is important for delineating dense crowd boundaries and spatially coherent regions. The depth-wise convolution branch compensates for this limitation by operating directly on the spatially arranged value features, providing the local neighborhood prior commonly emphasized in hierarchical vision Transformers [12].

Specifically, the value tensor is reshaped from the token sequence into the spatial grid as

(10) $${\bar{V}}_{\mathrm{spa}}^{\left(l\right)}=\mathrm{Reshape}\;\left(V_{\mathrm{spa}}^{\left(l\right)}\right)\in R^{B\times T\times H_{lr}\times W_{lr}\times d},$$

which is then permuted and flattened along the batch and temporal dimensions,

(11) $${\tilde{V}}_{\mathrm{spa}}^{\left(l\right)}=\mathrm{Reshape}\;\left(\mathrm{Permute}\;\left({\bar{V}}_{\mathrm{spa}}^{\left(l\right)}\right)\right)\in R^{\left(B\cdot T\right)\times d\times H_{lr}\times W_{lr}}.$$

Here, Permute(·) reorders the axes as $\left(B,\;T,\;H_{lr},\;W_{lr},\;d\right)\to \left(B,\;T,\;d,\;H_{lr},\;W_{lr}\right)$ before merging the batch and temporal dimensions. A depth-wise convolution is then applied on this grid representation,

(12) $$V_{\mathrm{dw}}^{\left(l\right)}=\mathrm{DWConv}\;\left({\tilde{V}}_{\mathrm{spa}}^{\left(l\right)}\right),$$

and the convolved features are reshaped back to the token form,

(13) $$Z_{\mathrm{dw}}^{\left(l\right)}={\mathrm{Reshape}}_{\mathrm{token}}\;\left(V_{\mathrm{dw}}^{\left(l\right)}\right)\in R^{B\times T\times N\times d},$$

where the inverse reshape uses the identity $N=H_{lr}W_{lr}$ to restore the spatial grid into the token sequence. The final spatial update is therefore given by

(14) $$Z_{\mathrm{spa}}^{\left(l\right)}={\hat{Z}}_{\mathrm{spa}}^{\left(l\right)}+Z_{\mathrm{dw}}^{\left(l\right)}.$$

This additive fusion allows the attention pathway and the convolutional pathway to provide complementary spatial cues, namely global context and local structural continuity. The resulting representation $Z_{\mathrm{spa}}^{\left(l\right)}$ thus encodes both long-range spatial dependencies and short-range local structures, and is subsequently used as the input to temporal attention.

After spatial aggregation, temporal attention is applied across frames for each spatial token:

(15) $${\tilde{Z}}_{\mathrm{spa}}^{\left(l\right)}=\mathrm{LN}\;\left(Z_{\mathrm{spa}}^{\left(l\right)}\right),$$

(16) $$Z_{\mathrm{tmp}}^{\left(l\right)}={\mathrm{MSA}}_{\mathrm{tmp}}\;\left({\tilde{Z}}_{\mathrm{spa}}^{\left(l\right)}\right)+Z_{\mathrm{spa}}^{\left(l\right)}.$$

This operation models the temporal evolution of each spatial region while preserving the spatial topology established by the preceding localized spatial aggregation.

Finally, a position-wise feed-forward network is applied:

(17) $$Z^{\left(l\right)}=\mathrm{FFN}\;\left(\mathrm{LN}\;\left(Z_{\mathrm{tmp}}^{\left(l\right)}\right)\right)+Z_{\mathrm{tmp}}^{\left(l\right)}.$$

These sequential operations yield the updated spatio-temporal representation $Z^{\left(l\right)}$ for the CoarseSTFormer block.

#### 4.1.2. Motion-Adaptive Spatial Anchor

Although the above factorized attention efficiently captures spatial and temporal dependencies, the resulting representation may still be insufficiently aligned under highly non-stationary crowd dynamics. In particular, simple temporal averaging tends to act as a low-pass filter, suppressing abrupt yet semantically important variations caused by sudden directional changes, local compression, or partial occlusions.

To preserve such dynamic cues, we construct a motion-adaptive spatial anchor from the final block output. We first estimate frame-wise temporal variation from the spatially attended features:

(18) $$\Delta Z_{t}=\left\{\begin{array}{cc} 0, & t=1,\\ \tanh\;\left(\left(Z_{\mathrm{spa},t}^{\left(L\right)}-Z_{\mathrm{spa},t-1}^{\left(L\right)}\right)W_{\Delta }\right), & t=2,\ldots ,T, \end{array}\right.$$

where $W_{\Delta }\in R^{d\times d}$ is a learnable projection matrix, $\Delta Z_{t}\in R^{B\times N\times d}$, and tanh(·) stabilizes the magnitude of temporal differences to prevent gradient explosion.

Next, we compute motion-aware temporal weighting coefficients conditioned on both the motion signal and the current spatial feature:

(19) $$\beta_{t}=\left[\Delta Z_{t}\;∥\;Z_{\mathrm{spa},t}^{\left(L\right)}\right]W_{\beta },\;\beta_{t}\in R^{B\times N\times 1},$$

(20) $$w_{t}={\mathrm{Softmax}}_{t}\left(\beta_{t}\right)=\frac{\exp(\beta_{t})}{\sum_{\tau =1}^{T}\exp\left(\beta_{\tau }\right)},\;w_{t}\in R^{B\times N\times 1},$$

where ‖ denotes channel-wise concatenation along the last axis (yielding a tensor in $R^{B\times N\times 2d}$), $W_{\beta }\in R^{2d\times 1}$ is a learnable projection matrix, and the softmax is applied explicitly along the temporal dimension (*t*).

Using these weights, the spatial anchor is formed as a temporally weighted aggregation of the temporally refined features:

(21) $$A_{\mathrm{spatial}}=\sum_{t=1}^{T}w_{t}⊙Z_{\mathrm{tmp},t}^{\left(L\right)},\;A_{\mathrm{spatial}}\in R^{B\times N\times d},$$

where ⊙ denotes element-wise multiplication with broadcasting along the channel dimension. Here, the temporal saliency weights $w_{t}$ are derived from the spatial branch output $Z_{\mathrm{spa},t}^{\left(L\right)}$ to capture spatially grounded motion variation, while the aggregation is applied to the temporally refined features $Z_{\mathrm{tmp},t}^{\left(L\right)}$ so that the resulting anchor inherits the temporal context modeled by the temporal attention. This anchor emphasizes dynamically salient regions while suppressing temporally redundant background responses, thereby serving as a robust sparsity-aware spatial prior for Stage 2 refinement. Conceptually, this spatial anchor can be interpreted as a motion-aware global pooling operator that preserves temporally salient spatial structures. Unlike standard attention pooling, it explicitly incorporates frame-to-frame temporal variation through $\Delta Z_{t}$, thereby improving sensitivity to non-stationary crowd dynamics.

#### 4.1.3. Feature Aggregation and Stage Output

After *L* stacked CoarseSTFormer blocks, the final low-resolution spatio-temporal representation is obtained as

(22) $$Z^{\left(L\right)}\in R^{B\times T\times N\times d}.$$

We reshape the token sequence back into the spatial grid to construct the coarse spatio-temporal feature tensor

(23) $$F_{\mathrm{global}}\in R^{B\times T\times d\times H_{lr}\times W_{lr}},$$

and similarly reshape the motion-adaptive spatial anchor $A_{\mathrm{spatial}}$ into the spatial grid using the identity $N=H_{lr}W_{lr}$, yielding

(24) $$A_{\mathrm{prior}}\in R^{B\times d\times H_{lr}\times W_{lr}}.$$

Unlike conventional approaches that treat such representations merely as intermediate features, we explicitly interpret $F_{\mathrm{global}}$ and $A_{\mathrm{prior}}$ as a *coarse-level predictive field* that encodes the likelihood of density-critical regions across space and time.

Specifically, the combination of globally aggregated context $F_{\mathrm{global}}$ and motion-aware spatial emphasis $A_{\mathrm{prior}}$ enables the model to highlight regions that are likely to exhibit high crowd density or rapid density transitions. This formulation allows Stage 1 to function not only as a feature extractor but also as an implicit region prioritization module, where spatial grids are softly organized according to their dynamic importance.

In summary, Stage 1 captures global crowd dynamics at a coarse resolution while preserving localized density structures and non-stationary temporal variations. More importantly, it produces a structured representation that anticipates where density concentration is likely to occur. This allows the subsequent stage to operate in a targeted manner, focusing computational resources on density-critical regions rather than uniformly processing the entire spatial domain. Consequently, this biases representation learning toward informative regions, thereby alleviating the tendency for easy background samples to dominate optimization, as discussed in our motivation.

### 4.2. Stage 2: SparseQueryDecoder—Sparse High-Resolution Response Reconstruction

**Figure 4:**
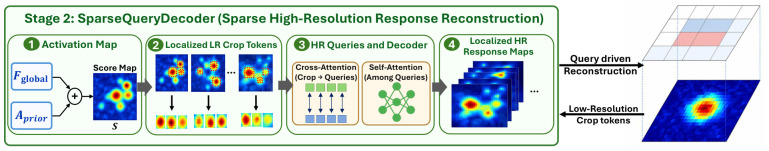
Detailed architecture of Stage 2 (SparseQueryDecoder).

Stage 1 produces a coarse-level predictive field, namely the global feature tensor $F_{\mathrm{global}}$ and the motion-adaptive spatial prior $A_{\mathrm{prior}}$, which jointly indicate density-critical candidate regions at low resolution. Rather than uniformly refining the entire spatial domain, Stage 2 focuses computation only on these active regions and performs localized high-resolution reconstruction in a coarse-to-fine manner. The detailed architecture of SparseQueryDecoder is illustrated in Figure 4.

To explicitly determine the active regions, we first construct a low-resolution activation logit map

(25) $$L_{S}=\mu_{g}\;\psi_{l}\left(F_{\mathrm{global}}\right)+\mu_{a}\;\varphi_{l}\left(A_{\mathrm{prior}}\right)\in R^{B\times 1\times H_{lr}\times W_{lr}},$$

where $\psi_{l}\left(\cdot \right)$ aggregates the temporal dimension of $F_{\mathrm{global}}$ via temporal mean pooling and then applies a 1×1 convolution, $\varphi_{l}\left(\cdot \right)$ applies a 1×1 convolution directly to $A_{\mathrm{prior}}$, and $\mu_{g},\mu_{a}$ are learnable balancing coefficients. The activation score map used for candidate ranking is then obtained as

(26) $$S=\sigma (L_{S}).$$

The Stage 1 score map is directly supervised at the low-resolution grid level. Specifically, the high-resolution density-critical ground-truth map is projected onto the low-resolution grid to obtain an active-cell label $Y_{lr}\in {\left\{0,1\right\}}^{H_{lr}\times W_{lr}}$, and the activation logit map is optimized using a binary cross-entropy-with-logits objective:

(27) $$L_{S1}=\mathrm{BCEWithLogits}\left(L_{S},Y_{lr}\right).$$

Thus, Stage 1 receives its own coarse-level supervision and is not learned only implicitly through the Stage 2 reconstruction loss.

Spatial locations with high activation scores are treated as candidate centers. To avoid redundant overlapping crops, non-maximum suppression is applied over *S*, and a fixed number of top-scoring locations are retained as active regions for each sample. This candidate-selection step is not included in the differentiable back-propagation path. NMS and top-*M* retention are discrete routing operations that convert the trained score map into crop indices; they are not treated as differentiable layers and are not used as paths for loss back-propagation. During training, the SparseQueryDecoder is optimized on pre-extracted localized crop instances paired with their corresponding high-resolution crop labels, using the focal-modulated objective described in Section 4.2.2. During inference, the trained Stage 1 score map is used to rank candidate locations, after which NMS and top-*M* retention select the crops to be refined by Stage 2.

For each of the *M* selected centers per sample, we extract a localized crop from the corresponding low-resolution input region, yielding

(28) $$X_{lr}\in R^{B\times M\times T\times C\times k_{lr}\times k_{lr}},$$

where *M* denotes the number of active regions per sample and $k_{lr}$ denotes the spatial crop size on the low-resolution grid. This explicit selection process establishes a reproducible interface between Stage 1 region prioritization and Stage 2 local refinement.

For notational simplicity, we treat each crop as an independent instance by merging the batch and region axes, $\left(B,M\right)\to B^{\prime }=B\cdot M$, so that all subsequent operations are described per crop. Specifically, each crop is reshaped by merging the spatial dimensions as $k_{lr}\times k_{lr}\to n_{lr}$ and concatenating the temporal and channel dimensions as (T,C)→T·C, yielding a set of localized low-resolution tokens

(29) $$Z_{lr}\in R^{B^{\prime }\times n_{lr}\times \left(T\cdot C\right)},\;n_{lr}=k_{lr}^{2},$$

where each token corresponds to one spatial location in the low-resolution crop, and its feature vector concatenates evidence from all *T* frames along the channel axis, providing temporally aware multi-channel inputs to the subsequent projection layer. These tokens are projected into the latent space by a learnable linear mapping,

(30) $$E_{lr}^{\left(0\right)}=Z_{lr}W_{\mathrm{proj}}+P_{lr},\;E_{lr}^{\left(0\right)}\in R^{B^{\prime }\times n_{lr}\times d},$$

where $W_{\mathrm{proj}}\in R^{(T\cdot C)\times d}$ is a learnable projection matrix and $P_{lr}\in R^{n_{lr}\times d}$ is a learnable positional embedding broadcast over the leading $B^{\prime }$ axis. To further encode local spatial context, we apply a lightweight Transformer encoder,

(31) $$E_{lr}=F_{enc}\;\left(E_{lr}^{\left(0\right)}\right)\in R^{B^{\prime }\times n_{lr}\times d},$$

so that $E_{lr}$ serves as a localized contextual memory for high-resolution reconstruction.

Let the target high-resolution crop size be $k_{hr}\times k_{hr}$, and define

(32) $$n_{hr}=k_{hr}^{2}.$$

We introduce a set of learnable high-resolution queries

(33) $${\bar{Q}}_{hr}\in R^{n_{hr}\times d},$$

where each query corresponds to one target location in the high-resolution crop, together with a learnable positional embedding $P_{hr}\in R^{n_{hr}\times d}$. These are shared across all crops and broadcast over the $B^{\prime }$ axis to form the initial query tensor,

(34) $$Q_{hr}^{\left(0\right)}={\mathrm{Broadcast}}_{B^{\prime }}\;\left({\bar{Q}}_{hr}+P_{hr}\right)\in R^{B^{\prime }\times n_{hr}\times d}.$$

#### 4.2.1. Query-Driven Conditional Reconstruction

The central idea of Stage 2 is to reconstruct each high-resolution target location not by uniformly expanding coarse features, but by allowing each target query to selectively retrieve only the contextual evidence relevant to its own position. Concretely, at the *l*-th decoder block, each high-resolution query first attends to the encoded low-resolution crop through cross-attention:

(35) $${\tilde{Q}}_{hr}^{\left(l\right)}=\mathrm{CrossAttn}\;\left(Q_{hr}^{\left(l-1\right)},E_{lr},E_{lr}\right)+Q_{hr}^{\left(l-1\right)}.$$

Using the standard attention formulation, this operation can be written as

(36) $$\mathrm{CrossAttn}\;\left(Q_{hr}^{\left(l-1\right)},E_{lr},E_{lr}\right)=\mathrm{Softmax}\;\left(\frac{\left(Q_{hr}^{\left(l-1\right)}W_{Q}^{\mathrm{dec},(l)}\right){\left(E_{lr}W_{K}^{\mathrm{dec},(l)}\right)}^{⊤}}{\sqrt{d}}\right)\left(E_{lr}W_{V}^{\mathrm{dec},(l)}\right),$$

where $W_{Q}^{\mathrm{dec},(l)},W_{K}^{\mathrm{dec},(l)},W_{V}^{\mathrm{dec},(l)}\in R^{d\times d}$ are learnable projection matrices specific to the decoder block, and are independent of the encoder-side projections used in Stage 1. This mechanism enables content-adaptive information transfer from a compact low-resolution context set to dense high-resolution target queries, thereby providing a non-uniform alternative to interpolation-based upsampling.

The updated queries are then refined through self-attention in the target space:

(37) $${\hat{Q}}_{hr}^{\left(l\right)}=\mathrm{SelfAttn}\;\left({\tilde{Q}}_{hr}^{\left(l\right)}\right)+{\tilde{Q}}_{hr}^{\left(l\right)}.$$

A position-wise feed-forward transformation is subsequently applied,

(38) $$Q_{hr}^{\left(l\right)}=\mathrm{FFN}\;\left({\hat{Q}}_{hr}^{\left(l\right)}\right)+{\hat{Q}}_{hr}^{\left(l\right)},$$

so that neighboring target locations can maintain structural consistency while the localized query representations are progressively refined.

After $L_{dec}$ decoder blocks, the final high-resolution query representation is given by

(39) $$Q_{hr}^{\left(L_{dec}\right)}\in R^{B^{\prime }\times n_{hr}\times d}.$$

A linear prediction head maps each decoded query to a pixel-wise logit,

(40) $$z=\mathrm{Head}\;\left(Q_{hr}^{\left(L_{dec}\right)}\right)\in R^{B^{\prime }\times n_{hr}\times 1},$$

where each entry $z_{i}$ corresponds to the logit at the *i*-th high-resolution location. Squeezing the trailing singleton dimension and reshaping to the target grid yields the localized high-resolution logit map

(41) $${\hat{L}}_{hr}\in R^{B^{\prime }\times k_{hr}\times k_{hr}},$$

which is subsequently passed through a sigmoid activation during inference to obtain the corresponding response probability map, and is directly used as the logit input for the focal-modulated optimization described in the next subsection.

Overall, the SparseQueryDecoder performs localized high-resolution reconstruction by combining content-adaptive context retrieval from low-resolution crop tokens with structural refinement among target queries. As a result, Stage 2 enables sparse and targeted high-resolution reconstruction over density-critical regions identified in Stage 1, rather than expending computation uniformly over the entire spatial domain.

#### 4.2.2. Focal-Modulated Optimization Under Extreme Local Sparsity

Even after restricting reconstruction to active local crops, the target high-resolution response map remains highly imbalanced. Within each crop, only a small subset of pixels typically corresponds to density-critical responses, whereas the majority remain background. Under standard Binary Cross-Entropy (BCE) optimization, gradients from these easy negative pixels can dominate training, making it difficult for the model to accurately localize rare high-response regions.

To address this issue, we adopt a focal-modulated BCE objective [27]. Let $z_{i}$ denote the predicted logit for the *i*-th pixel in the high-resolution crop, and let

(42) $$p_{i}=\sigma \left(z_{i}\right)$$

be the corresponding predicted probability, where $y_{i}\in \left\{0,1\right\}$ denotes the binary ground-truth response label. The pixel-wise BCE loss is defined as

(43) $$L_{\mathrm{BCE}}^{\left(i\right)}=-\left[y_{i}\log p_{i}+\left(1-y_{i}\right)\log\left(1-p_{i}\right)\right].$$

We further define the target-aligned confidence

(44) $$p_{i}^{t}=y_{i}p_{i}+\left(1-y_{i}\right)\left(1-p_{i}\right),$$

which measures the confidence assigned to the correct class. Using this quantity, the focal-modulated loss for the *i*-th pixel is

(45) $$L_{\mathrm{focal}}^{\left(i\right)}=\alpha_{i}{\left(1-p_{i}^{t}\right)}^{\gamma }L_{\mathrm{BCE}}^{\left(i\right)},$$

where γ is the focusing parameter and $\alpha_{i}$ is the class-balancing coefficient:

(46) $$\alpha_{i}=\left\{\begin{array}{cc} \alpha , & y_{i}=1,\\ 1-\alpha , & y_{i}=0. \end{array}\right.$$

The final localized training objective is defined as

(47) $$L_{\mathrm{total}}=\frac{1}{n_{hr}}\sum_{i=1}^{n_{hr}}\left[\lambda L_{\mathrm{BCE}}^{\left(i\right)}+\left(1-\lambda \right)L_{\mathrm{focal}}^{\left(i\right)}\right].$$

This objective suppresses gradients from well-classified background pixels while preserving strong supervision on hard positive responses. Consequently, it biases optimization toward rare but important density-critical regions, which is essential for accurate localized reconstruction under extreme spatial sparsity.

### 4.3. Computational Complexity and Hardware-Efficient Sparse Inference

A key limitation of conventional high-resolution crowd density models is that they perform dense computation over the entire spatial domain, even though severe congestion events typically occupy only a small fraction of the scene. Let

(48) $$N_{hr}=H_{hr}W_{hr}$$

denote the number of spatial tokens in the full high-resolution space, let *T* be the number of frames, and let *d* be the hidden dimension. A naive global spatio-temporal transformer operating directly on the full-resolution video incurs a complexity of

(49) $$C_{\mathrm{global}}=O\;\left({\left(TN_{hr}\right)}^{2}d\right)=O\;\left(T^{2}N_{hr}^{2}d\right),$$

since all pairwise interactions are computed jointly over the entire spatio-temporal token set.

The proposed framework avoids this cost by decomposing inference into a coarse global screening stage and a sparse local refinement stage. In Stage 1, the input is processed at a reduced spatial resolution with downsampling factor *s* so that the effective number of spatial tokens becomes

(50) $$N_{lr}=\frac{N_{hr}}{s^{2}}.$$

Because Stage 1 employs factorized spatio-temporal attention rather than joint spatio-temporal attention, its dominant complexity becomes

(51) $$C_{S1}=O\;\left(TN_{lr}^{2}d+N_{lr}T^{2}d\right)=O\;\left(T\frac{N_{hr}^{2}}{s^{4}}d+\frac{N_{hr}}{s^{2}}T^{2}d\right).$$

Thus, Stage 1 preserves global spatio-temporal reasoning while substantially reducing the cost relative to dense full-resolution processing.

Rather than directly reconstructing the full high-resolution map, Stage 2 is activated only on candidate regions identified by Stage 1. Let *M* denote the number of activated local regions, let $n_{lr}$ be the number of low-resolution tokens in each local crop, let $n_{hr}$ be the number of target high-resolution queries per crop, and let $L_{dec}$ denote the number of decoder blocks. The complexity of Stage 2 is then

(52) $$C_{S2}=O\;\left(ML_{dec}\left(n_{hr}n_{lr}d+n_{hr}^{2}d\right)\right),$$

where the first term corresponds to cross-attention between high-resolution queries and low-resolution crop tokens, and the second term corresponds to self-attention within the target query set.

The total complexity of the proposed framework is therefore

(53) $$C_{\mathrm{proposed}}=C_{S1}+C_{S2}.$$

Defining the active high-resolution coverage ratio as

(54) $$\rho =\frac{Mn_{hr}}{N_{hr}},$$

we note that realistic crowd scenes typically satisfy ρ≪1, since density-critical events are sparse across the scene. Under this condition, the proposed framework avoids dense full-resolution refinement and instead allocates high-resolution computation only to a small subset of spatial regions.

This decomposition also reduces the effective inference-time activation footprint. In dense full-resolution processing, intermediate activations scale with the full token set,

(55) $$M_{\mathrm{global}}=\Theta \left(N_{hr}d\right),$$

whereas sparse local refinement requires

(56) $$M_{S2}=\Theta \left(Mn_{hr}d\right).$$

Therefore, beyond lowering arithmetic complexity, the proposed two-stage design also reduces the amount of high-resolution feature storage and movement required during inference.

Overall, the proposed architecture improves inference efficiency in two complementary ways. Stage 1 performs global spatio-temporal screening at low resolution, thereby avoiding expensive dense reasoning over the full high-resolution token space, while Stage 2 concentrates high-resolution decoding only on regions that are likely to contain density-critical responses. This coarse-to-fine sparse inference strategy provides a substantially more hardware-efficient alternative to conventional dense high-resolution processing.

## 5. Simulation Results

### 5.1. Simulation Setups

To evaluate the proposed framework under controlled yet large-scale crowd dynamics, we constructed a synthetic pedestrian simulation environment [28] over a 200×200 2D world. Within this environment, crowd movement was simulated under multiple population scales in order to examine the robustness of the model across varying congestion levels. Specifically, five crowd-density settings were considered, containing 10,000, 12,000, 14,000, 16,000, and 20,000 pedestrian agents.

Each agent was assigned an individual physical radius sampled from the range [0.2,0.7], thereby introducing variability in agent size and local occupancy. The walking speed *v* of each agent was sampled from Gaussian distributions defined within the range [0.8,2.0], allowing the simulation to reflect heterogeneous motion characteristics across different pedestrians [29]. Initial movement directions were randomly assigned.

All simulations were executed on a workstation equipped with an AMD EPYC 9354 32-Core Processor (64 logical CPUs), 251 GB system memory, and a single NVIDIA L40S GPU with 46 GB memory. The resulting simulated trajectories and density patterns were converted into video-like spatio-temporal inputs for model training and evaluation.

The data for each grid scale were generated as an independent simulation set, yielding 12,400 scenes for the small grid and 5843 scenes each for the medium and large grids, with every set split 85/15 into training and held-out validation under a fixed random seed. Each scene pairs an 8-frame observation window, sampled at a 5-second interval, with a future ground-truth frame at the target horizon so that the prediction horizons of 2, 5, and 10 frames correspond to 10, 25, and 50 seconds ahead. The bird’s-eye-view inputs are formed at low resolutions of 40×40, 20×20, and 14×14 for the small, medium, and large grids and reconstructed at a common 200×200 high resolution. Ground-truth density-critical labels are obtained by Gaussian-smoothing the agent positions (σ=2.0 m) and thresholding the resulting density at 5.0 person/m^2^. All models are trained with AdamW (learning rate $10^{-3}$, weight decay $10^{-4}$) and a plateau-based learning-rate scheduler for up to 100 epochs at a batch size of 32, using an embedding dimension of 128 and four blocks in each stage.

### 5.2. Performance of CoarseSTFormer

#### 5.2.1. Impact of Crowd Volume on Coarse Localization

**Figure 5:**
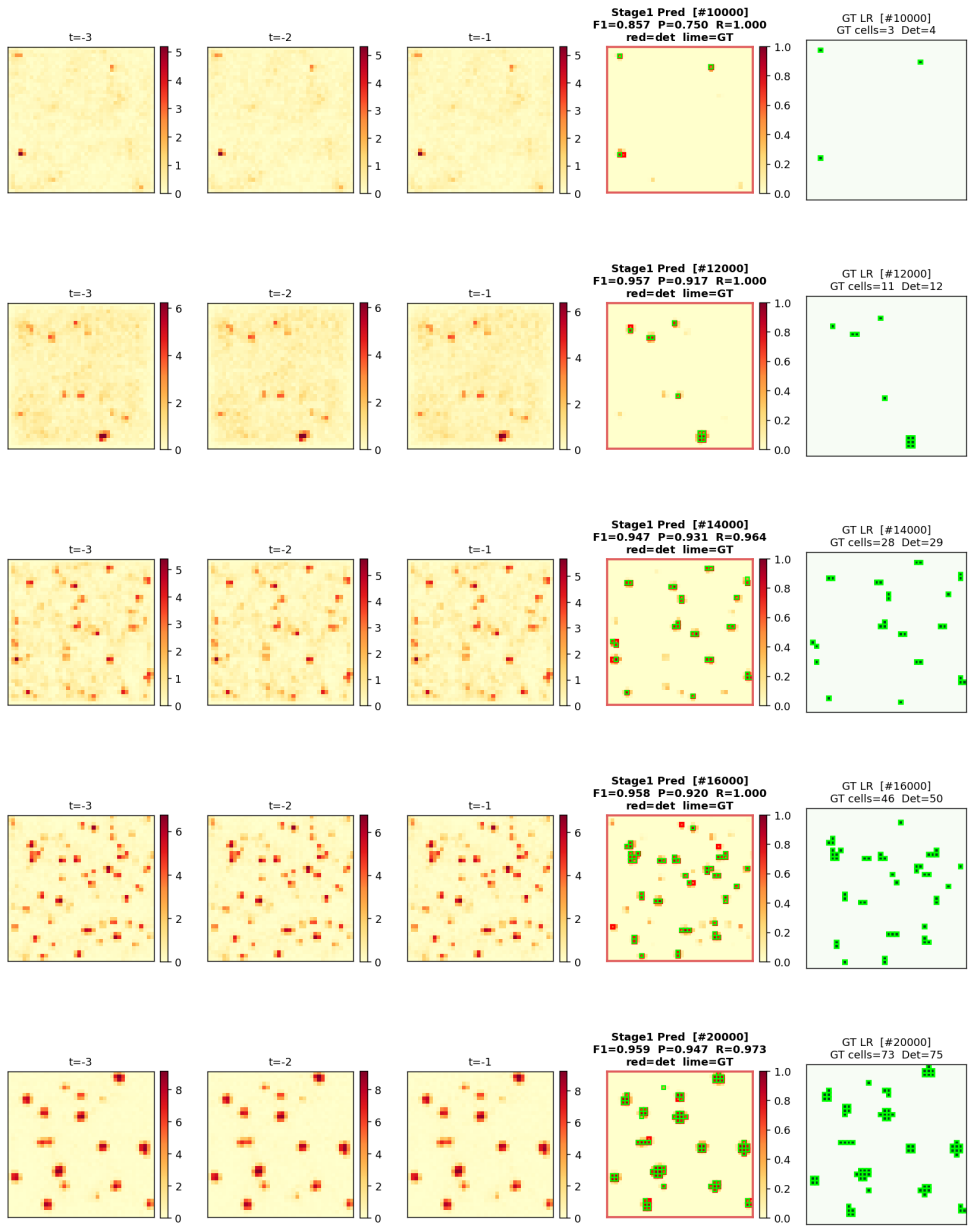
Representative Stage 1 inference results of CoarseSTFormer under different crowd densities. From the top row to the bottom row, the results correspond to simulations with 10,000, 12,000, 14,000, 16,000, and 20,000 agents, respectively. In the prediction column, red and green boxes denote cells detected by Stage 1 and ground-truth density-critical cells, respectively.

To analyze the role of CoarseSTFormer, we examine its Stage 1 localization under progressively denser crowds. The objective of Stage 1 is not the final high-resolution map but a reliable coarse activation field marking density-critical candidates for refinement. Its error structure is asymmetric: a missed cell cannot be recovered by Stage 2, whereas a redundant one is filtered during refinement. Stage 1 should therefore favor recall while keeping precision controlled. We report this behavior over the 646 active validation scenes, with every cell decided at a fixed threshold τ=0.5 so that the ground-truth, detected, and true-positive (TP) counts stay consistent with the reported $\left(P,\;R,\;F_{1}\right)$ in every row. The scenes are grouped into five density bins by ground-truth active-cell count and labeled #10000 through #20000 to denote increasing congestion. Representative Stage 1 inference maps for these five settings are shown in Figure 5.

Table 2 summarizes the aggregate behavior, from which two consistent patterns emerge. First, recall exceeds precision in every bin, and the mean number of detected cells is slightly larger than the mean number of ground-truth cells; for the #16000 setting, for instance, recall reaches 0.967 against a precision of 0.946, with 41.7 detected cells against 40.7 ground-truth cells on average. This is precisely the recall-favoring behavior expected of a screening module that may over-include candidate cells but must not discard critical ones. Second, the variance of all three metrics decreases steadily as the crowd grows denser. In the sparsest setting, the active-cell set is so small that minor absolute differences translate into large fluctuations in $F_{1}$; missing one of three ground-truth cells alone shifts $F_{1}$ by more than 0.10, which accounts for the elevated standard deviation of 0.239. The corresponding mean of 0.874 nonetheless remains well above the regime of diffuse background activation, indicating that the larger spread reflects the discreteness of few-cell scenes rather than any degradation of the underlying localization. As the crowd structure becomes more spatially pronounced, the variance contracts by nearly an order of magnitude to 0.023 in the densest setting, and the coarse activation field aligns increasingly tightly with the ground-truth distribution, with predicted cells concentrating around meaningful congestion hotspots rather than spreading across the background.

The aggregate view captures the dominant trend, but a single-scene view is useful for two further reasons. It makes the underlying computation fully transparent, since presenting the (GT, Det, TP) triple of one scene at a fixed threshold leaves no ambiguity about how the reported scores are obtained. It also confirms that the recall-favoring tendency is a genuine property of the trained module rather than an averaging effect that might emerge only when recall-favoring and precision-favoring scenes cancel out. Table 3 therefore reports one representative scene per crowd-volume bin, selected as the scene whose $F_{1}$ lies near the bin mean rather than at the perfect-localization mode that dominates the sparsest bin, with the TP column shown explicitly so that every row remains internally consistent.

The per-scene results in Table 3 reproduce the same pattern at every density level. In the sparsest example, the model retains all three ground-truth cells at perfect recall while activating a single additional cell, yielding a precision of 0.750 and an $F_{1}$ of 0.857; this is the over-include-but-never-miss behavior expected in a regime where the loss of even one cell would forfeit a substantial fraction of $F_{1}$. The #12000 scene preserves perfect recall by detecting twelve cells against eleven ground-truth cells, with a precision of 0.917 and an $F_{1}$ of 0.957, and the relative safety margin narrows as density increases. The same tendency persists into the denser cases, where CoarseSTFormer attains $\left(P,\;R,\;F_{1}\right)$ of (0.931, 0.964, 0.947), (0.920, 1.000, 0.958), and (0.947, 0.973, 0.959) for #14000, #16000, and #20000, respectively. Throughout, the detected set exceeds the ground-truth set by at most a few cells, recall stays at or near unity, and the modest precision gap constitutes the safety margin that prevents Stage 2 from losing density-critical regions. Because this behavior appears identically in the aggregate and at the scene level, the recall-favoring bias is best understood as an intrinsic characteristic of the trained module.

The observed behavior is closely related to the internal design of CoarseSTFormer. The density-guided localized spatial attention suppresses the oversmoothing tendency of standard global attention and helps preserve compact responses around true congestion patterns. The depth-wise convolution branch reinforces short-range neighborhood consistency, which is important for maintaining coherent local structures in the low-resolution activation field. The motion-adaptive spatial anchor stabilizes temporal aggregation by emphasizing dynamically salient regions while suppressing temporally redundant background responses. The strong precision–recall trade-off observed in Table 2 and Table 3 therefore reflects the complementary interaction of these components, as localized spatial biasing improves the discrimination of candidate hotspots, local convolution sharpens spatial continuity, and motion-adaptive aggregation preserves non-stationary crowd cues over time.

Overall, these results verify that CoarseSTFormer fulfills its intended role in the proposed framework. It is neither an overly permissive candidate generator that activates excessive background regions, nor an overly selective filter that risks missing density-critical cells. Instead, it produces a reliable coarse activation field that preserves critical candidates with high recall while maintaining sufficient precision for efficient Stage 2 refinement.

#### 5.2.2. Impact of Prediction Horizon on Temporal Robustness

To further examine the temporal robustness of CoarseSTFormer, we evaluate Stage 1 under different prediction horizons. The prediction horizon indicates how far into the future the model predicts density-critical regions from the observed input sequence. We consider three representative settings: a short-term horizon of 2, a mid-term horizon of 5, and a long-term horizon of 10. This analysis is important because Stage 1 is expected to provide a reliable coarse activation field not only for near-future crowd states, but also for temporally distant and more uncertain density evolution.

In the proposed coarse-to-fine framework, prediction horizon has a direct influence on the difficulty of Stage 1 localization. When the horizon is short, future density-critical regions are strongly correlated with the most recent observations, and the model can exploit local temporal continuity. In contrast, as the horizon becomes longer, the future crowd distribution becomes increasingly uncertain due to accumulated motion variation, local interactions among agents, directional changes, and the possible emergence or disappearance of congestion regions. Therefore, long-horizon prediction requires the model to infer not only immediate motion continuity, but also temporally persistent spatial structures that remain predictive beyond the observed frames.

As summarized in Table 4, CoarseSTFormer maintains strong coarse localization performance across different temporal gaps. For the short-term prediction setting with horizon 2, the model achieves (P, R, F1)=(0.935, 0.960, 0.947). This result indicates that Stage 1 can accurately extrapolate near-future density-critical regions from the recent input sequence. The detected cell count is also close to the number of ground-truth active cells, with 77 detected cells against 75 ground-truth cells, showing that the model preserves most critical regions without substantially enlarging the candidate set.

For the mid-term prediction setting with horizon 5, the model achieves the strongest performance, with (P, R, F1)=(0.951, 0.970, 0.961). Despite the increased temporal gap, both precision and recall remain high, and the number of detected cells remains closely aligned with the ground truth, with 103 detected cells against 101 ground-truth cells. This suggests that CoarseSTFormer does not merely depend on short-range frame-to-frame similarity. Instead, it captures temporally stable crowd dynamics and spatially coherent density patterns that remain informative over a moderate future interval. In particular, the high recall demonstrates that density-critical regions are rarely missed, while the high precision indicates that the activation field remains compact rather than becoming overly permissive.

When the prediction horizon is extended to 10, the task becomes considerably more challenging. The model achieves (P, R, F1)=(0.798, 0.893, 0.843), showing a degradation compared with shorter horizons. This decrease is expected because long-term future density maps are inherently less deterministic: small differences in local motion can accumulate over time, and congestion patterns may shift, merge, or dissipate before the target frame. Nevertheless, the model still maintains a recall of 0.893, preserving the majority of future density-critical cells. From the perspective of the proposed two-stage framework, this behavior is particularly meaningful. Since Stage 1 serves as a coarse screening module, false negatives are more harmful than false positives: a missed active cell cannot be recovered by the high-resolution decoder, whereas an additional candidate can still be filtered or corrected during Stage 2 refinement. Therefore, the relatively recall-preserving behavior at horizon 10 indicates that CoarseSTFormer remains suitable for long-horizon candidate generation.

**Figure 6:**
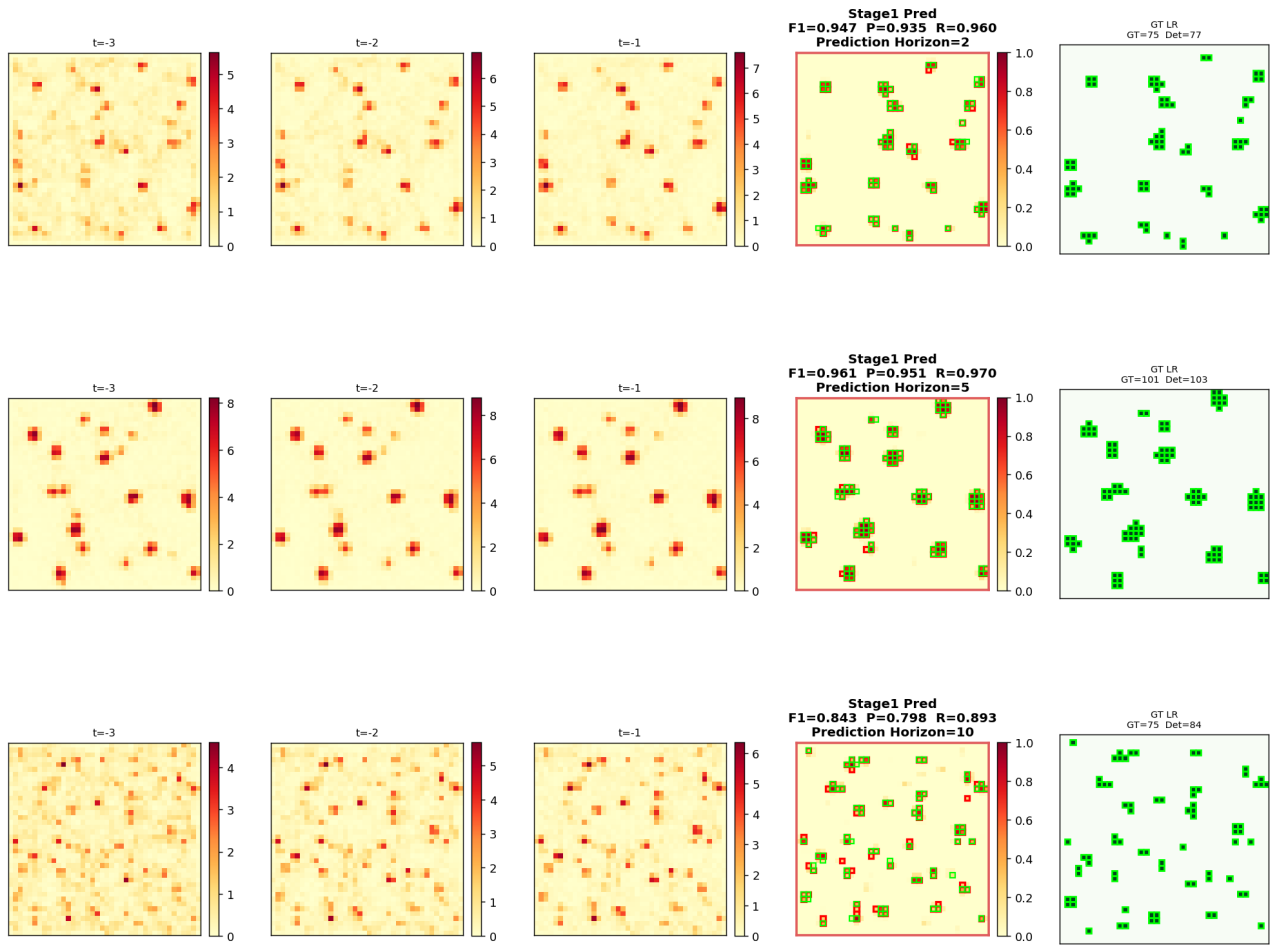
Representative Stage 1 inference results under different prediction horizons. From the top row to the bottom row, the results correspond to prediction horizons of 2, 5, and 10, respectively. In each row, the first three columns show the recent low-resolution input sequence, the fourth column shows the Stage 1 prediction, and the last column shows the corresponding low-resolution ground truth. CoarseSTFormer consistently preserves future density-critical regions across short-, mid-, and long-term prediction settings. Although long-horizon prediction introduces greater temporal uncertainty, the predicted active cells remain concentrated around meaningful future congestion candidates rather than spreading uniformly over the background. In the prediction column, red and green boxes denote cells detected by Stage 1 and ground-truth density-critical cells, respectively.

The qualitative results in Figure 6 further support this interpretation. For horizons 2 and 5, the predicted activation maps show strong spatial agreement with the future ground-truth cells, indicating that the recent input sequence contains sufficient temporal evidence for reliable short- and mid-term extrapolation. At horizon 10, the prediction becomes less sharply aligned, which is consistent with the reduction in precision. However, the degradation mainly appears as additional neighboring candidate activations rather than a complete loss of future congestion regions. This distinction is important: the model does not simply overactivate the entire background under temporal uncertainty, but instead expands the candidate field around plausible future congestion locations. Therefore, the long-horizon error pattern suggests conservative candidate expansion around plausible future congestion locations rather than random false activation. We note that this is a behavioral property of the trained module rather than an explicit estimate of predictive uncertainty; principled uncertainty modeling for localization tasks, as systematically reviewed in the mobile-crowdsensing positioning literature [30], is a complementary direction that we leave for future work.

This behavior provides a different perspective from the crowd-volume analysis. While the previous analysis examined whether Stage 1 remains reliable under changes in crowd density, the prediction-horizon analysis evaluates whether the coarse activation field remains temporally stable as the target time moves farther away from the observed sequence. The results show that performance degradation is gradual rather than abrupt. In particular, the recall remains relatively high even at horizon 10, indicating that the model preserves temporally persistent congestion cues despite accumulated motion uncertainty. This suggests that Stage 1 learns a coarse predictive field that is not limited to immediate frame-to-frame displacement, but instead captures broader crowd-flow tendencies that remain informative over longer temporal intervals.

Overall, increasing the prediction horizon primarily affects the sharpness of localization rather than the ability to identify future density-critical regions. The decrease in precision at horizon 10 reflects the inherent ambiguity of long-term crowd evolution, where multiple future locations may become plausible candidates. Nevertheless, the model continues to retain most true active regions, which is the desired behavior for a coarse screening stage. Thus, the prediction-horizon results demonstrate that CoarseSTFormer provides temporally robust candidate generation: as future uncertainty increases, it degrades conservatively by slightly broadening plausible candidate regions, rather than catastrophically missing critical regions or collapsing into diffuse background responses.

#### 5.2.3. Impact of Grid Resolution on Coarse Localization

To evaluate whether Stage 1 is robust to different spatial discretization settings, we analyze the coarse localization performance under multiple grid resolutions. In the proposed framework, the low-resolution grid defines the spatial unit used for candidate region selection before high-resolution refinement. Therefore, Stage 1 should not depend on a single grid configuration, but should consistently generate reliable candidate regions across different spatial granularities.

Changing the grid resolution affects the nature of coarse localization. Depending on the discretization scale, density-critical regions may appear as fragmented small active cells, merged coarse responses, or boundary-sensitive activation patterns. A finer spatial partition can increase localization sensitivity because small shifts near cell boundaries may change the active-cell assignment. Conversely, a coarser partition can aggregate nearby density responses, making candidate regions more compact but potentially reducing fine-grained spatial separability. Robust performance across these settings therefore indicates that the model is not merely tuned to a particular grid layout, but learns a stable coarse predictive field for density-critical region selection.

As summarized in Table 5, CoarseSTFormer achieves consistently strong coarse localization performance across the evaluated grid settings. For grid size 5, the model obtains (P, R, F1)=(0.891, 0.953, 0.921), with 46 detected cells against 43 ground-truth cells. This case contains a relatively larger number of active cells, making the activation field more spatially distributed. Nevertheless, the model preserves most ground-truth active regions while keeping the detected candidate set close to the target scale.

For grid size 10, CoarseSTFormer achieves (P, R, F1)=(0.929, 1.000, 0.963), detecting 14 cells against 13 ground-truth cells. The perfect recall indicates that all density-critical cells are preserved for subsequent high-resolution refinement, while the high precision shows that the candidate field remains compact. This result demonstrates that Stage 1 can maintain a favorable balance between candidate preservation and candidate compactness under a different spatial discretization setting.

For grid size 15, the model also maintains strong localization performance, achieving (P, R, F1)=(0.947, 1.000, 0.973) with 19 detected cells against 18 ground-truth cells. Although this case exhibits a different spatial distribution of active regions, CoarseSTFormer still produces a well-aligned coarse activation map. In particular, the detected cells remain concentrated around ground-truth active regions rather than being scattered across irrelevant background areas.

**Figure 7:**
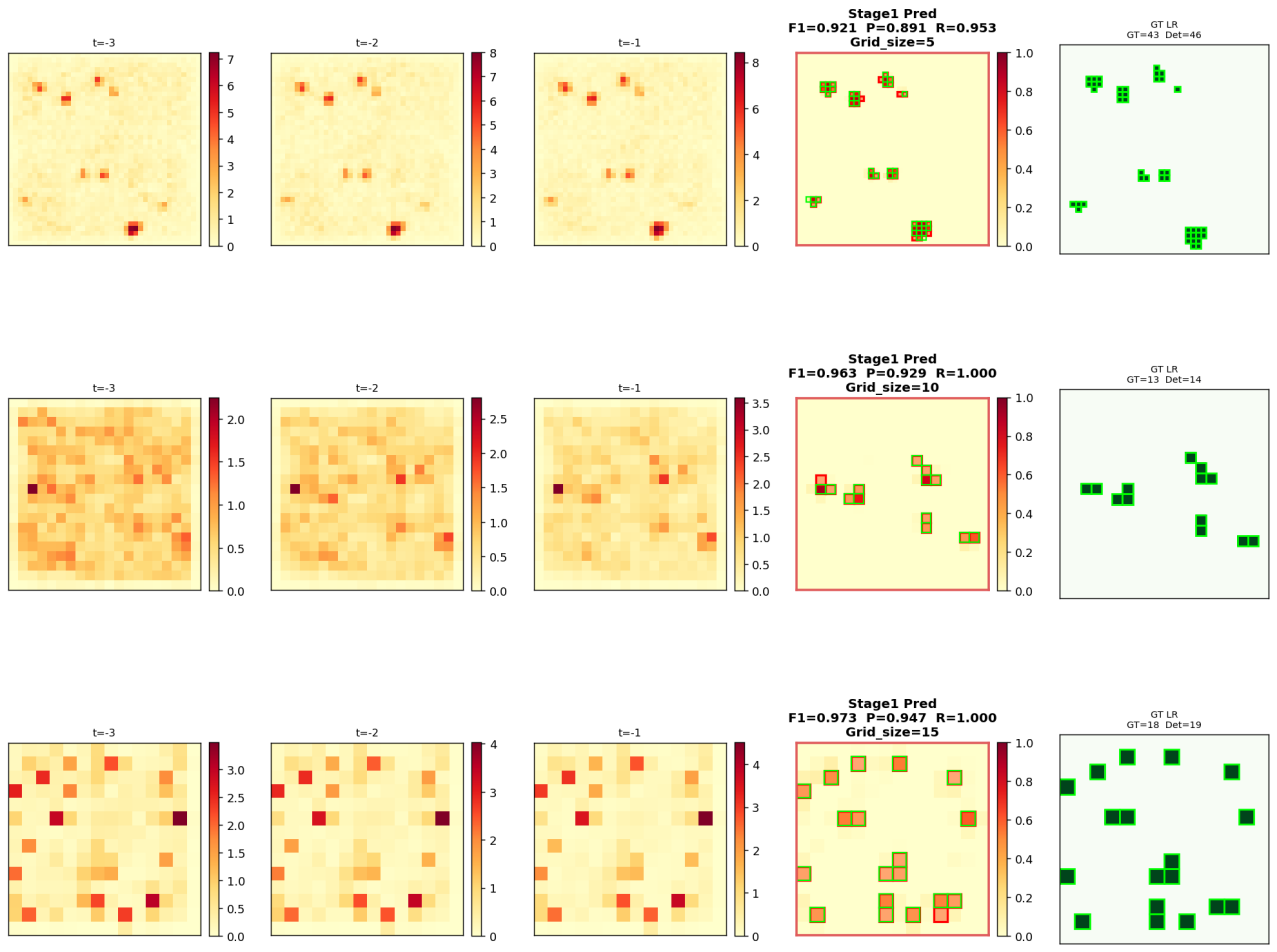
Representative Stage 1 inference results under different grid resolutions. From the top row to the bottom row, the results correspond to grid sizes of 5, 10, and 15, respectively. In each row, the first three columns show the recent low-resolution input sequence, the fourth column shows the Stage 1 prediction, and the last column shows the corresponding low-resolution ground truth. In the prediction column, red and green boxes denote cells detected by Stage 1 and ground-truth density-critical cells, respectively.

The qualitative results in Figure 7 further support this observation. Across the three representative cases, the predicted active regions follow the spatial structure of the ground-truth cells despite differences in grid scale, active-cell count, and hotspot arrangement. This suggests that the model’s localization behavior is not tied to a single spatial discretization pattern. Instead, Stage 1 adapts to different grid configurations by producing candidate fields that remain both recall-preserving and spatially compact.

Overall, these results indicate that CoarseSTFormer maintains reliable coarse localization across the evaluated grid configurations. Since the three cases are not controlled re-discretizations of the same scene, the results should be interpreted as evidence of generalization across heterogeneous grid configurations rather than as a direct resolution-scaling curve. Even under this broader evaluation setting, the model maintains high F1 scores and consistently high recall, indicating that Stage 1 can reliably identify density-critical candidate regions without requiring delicate tuning to a single fixed grid size. This property is important for the proposed coarse-to-fine framework, because the effectiveness of Stage 2 depends on receiving a stable and compact candidate set from Stage 1.

### 5.3. Performance of SparseQueryDecoder

#### 5.3.1. Impact of Grid Resolution on High-Resolution Reconstruction

To analyze the role of SparseQueryDecoder in the proposed coarse-to-fine framework, we examine the Stage 2 high-resolution reconstruction results under different grid resolutions. As discussed in Section 4, the objective of Stage 2 is not to revisit the entire spatial domain, but to take the compact candidate set produced by CoarseSTFormer and decode a fine-grained density response only on those density-critical regions. In this setting, the dominant difficulty is no longer candidate selection but reconstruction quality: the decoder must recover the spatial extent, boundary, and intensity of each congestion region from a coarse low-resolution activation rather than from the full pixel grid. Consequently, a desirable Stage 2 module should be robust to changes in grid resolution, since the spatial scale of a single coarse cell directly affects how much pixel-level structure must be inferred per query.

Changing the grid resolution alters the nature of Stage 2 refinement in two ways. First, the number of refined cells differs: a finer grid yields a larger candidate set with smaller per-cell coverage, whereas a coarser grid yields fewer candidates with larger per-cell coverage. Second, the relative spatial scale of a congestion blob within a single cell changes: in finer grids, a congestion region tends to span many adjacent cells and almost fully covers each one, whereas in coarser grids, a single cell may contain an entire compact congestion region surrounded by background.

As summarized in Table 6, SparseQueryDecoder achieves consistently strong whole-map reconstruction across the evaluated grid configurations, attaining (Dice, IoU) of (0.926, 0.862), (0.891, 0.804), and (0.897, 0.813) at s=5×, s=10×, and s=15×, respectively. Across all three settings, the number of active cells consistently exceeds the number of positive cells (e.g., 124 vs. 76 at s=5×); this over-selection is by design, since a missed positive cannot be recovered by Stage 2, and false-positive candidates are handled by the decoder’s learned background response. Despite an almost 5× variation in candidate cardinality across the three settings, the performance gap remains narrow, indicating that the decoder operates reliably without depending on a particular grid layout.

The whole-map scores do not degrade strictly monotonically with grid scale: the medium grid yields the lowest values, while the coarsest grid partially recovers. This non-monotonic pattern reflects how the per-cell reconstruction regime shifts with discretization, as further analyzed at the per-cell level in Table 7. The medium grid is the boundary-dominant regime, where each cell contains a compact blob surrounded by background and per-cell boundary contributions dominate the assembled map; the coarsest grid, by contrast, concentrates each region within a small portion of a mostly background cell, which the decoder localizes more easily and which partly offsets the larger per-cell footprint. Even with this non-monotonic behavior, the whole-map IoU stays within 5.8 percentage points across all three settings, confirming the decoder’s robustness across widely different per-cell responsibilities. The whole-map scores are also systematically lower than the per-cell scores; this gap is structural rather than indicative of stitching deficiency, since the whole-map metric accumulates boundary contributions and residual responses across all selected regions and the surrounding canvas, whereas the per-cell metric is restricted to a localized 3×3 context window around a single active cell.

**Figure 8:**
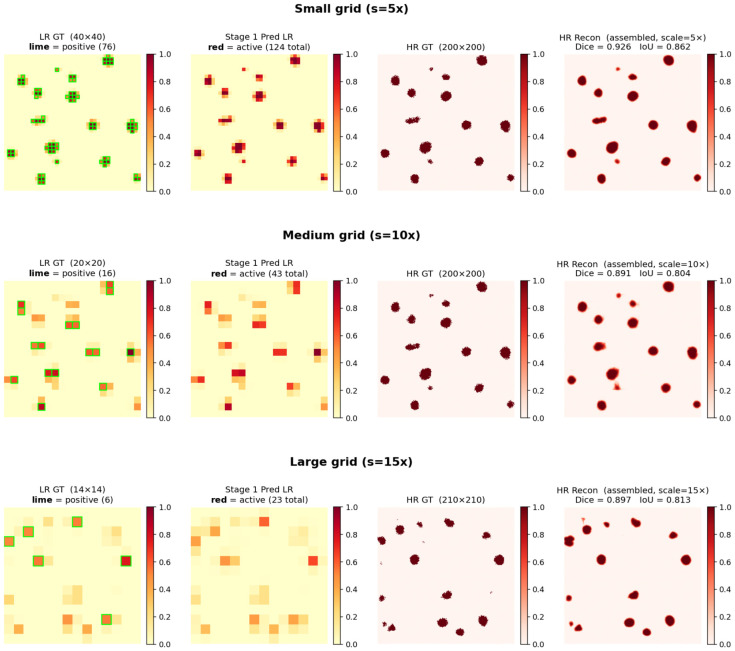
Representative whole-map Stage 2 reconstruction results under different grid resolutions. From the top row to the bottom row, the results correspond to grid scales s=5× (LR 40×40), s=10× (LR 20×20), and s=15× (LR 14×14), respectively. Green boxes (left) mark ground-truth positive cells; red boxes (second panel) mark the candidates selected by Stage 1.

Importantly, the qualitative results in Figure 8 show that the model’s robustness is not achieved by uniformly enlarging or shrinking responses: across all three rows, the reconstructed congestion regions remain spatially localized at the correct locations, with sharp boundaries that follow the ground-truth blobs rather than diffusing over the background. This behavior is closely tied to the internal design of SparseQueryDecoder. Because the high-resolution response is produced through learnable queries that attend back to the coarse feature representation, the decoder does not encode an absolute spatial prior tied to a specific grid size. Instead, each cell-level query learns to reconstruct the local high-resolution density conditioned on the corresponding coarse activation, regardless of how much spatial area that cell represents. Consequently, when the grid becomes coarser and each cell carries a larger physical footprint, the same query mechanism continues to operate, simply producing a larger but still correctly localized response. This scale-flexibility is what allows the model to maintain comparable Dice and IoU across s=5×, s=10×, and s=15× without grid-specific re-tuning.

#### 5.3.2. Per-Cell Reconstruction Fidelity Across Grid Resolutions

Although the whole-map metrics in Table 6 characterize the global reconstruction quality, they aggregate the contribution of every active cell together with the background, and therefore do not directly reveal how well SparseQueryDecoder reconstructs an individual congestion region. To examine this finer aspect, we additionally analyze the per-cell reconstruction quality on representative active cells from each grid scale, with each cell visualized together with one surrounding cell on every side (3×3 cell context window).

As summarized in Table 7, SparseQueryDecoder achieves consistently high per-cell Dice across all grid scales, with mean values of 0.960, 0.908, and 0.941 for s=5×, s=10×, and s=15×, respectively. Two observations are worth emphasizing. First, the per-cell scores are uniformly above 0.89, indicating that the local reconstruction quality is high regardless of how much spatial area a single cell represents. Second, the variance across the four representative cells within each grid is small (the maximum within-grid spread is at most 0.048), suggesting that the decoder does not rely on a few easy cases to produce its overall performance, but instead refines each candidate cell with comparable fidelity.

**Figure 9:**
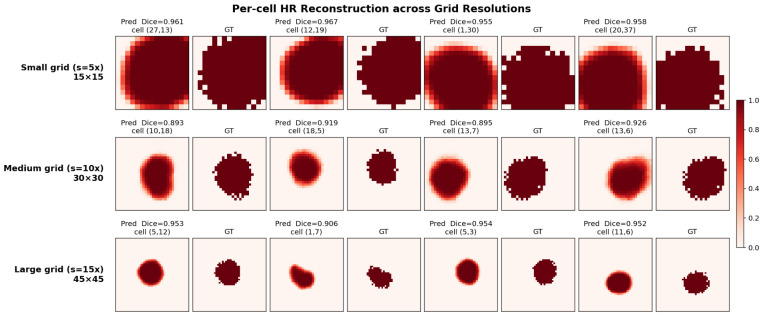
Representative per-cell high-resolution reconstruction results across grid resolutions. From the top row to the bottom row, the results correspond to grid scales s=5× (15×15 per-cell context), s=10× (30×30 per-cell context), and s=15× (45×45 per-cell context), respectively.

The relationship between the per-cell scores and the grid scale is informative. The finest grid (s=5×) yields the highest per-cell Dice (0.960), partly as a structural consequence of the regime: as shown in the top row of Figure 9, the 15×15 context is largely occupied by the congestion region, making the score less sensitive to boundary deviations. The medium grid (s=10×) presents a structurally more demanding regime, in which each 30×30 context contains a compact blob with non-trivial surrounding background; the decoder must simultaneously localize the blob, delineate its boundary, and assign low response to the background. The mean Dice of 0.908 in this regime demonstrates that SparseQueryDecoder performs genuine boundary-aware reconstruction rather than relying on foreground dominance.

The coarsest grid (s=15×) shows a notable recovery in per-cell Dice (0.941) compared with the medium grid. This is consistent with a different structural reason. In the 45×45 per-cell context, the congestion region appears as a smaller compact blob within a larger background area, so the foreground occupies only a small fraction of the patch. In this regime, the absolute foreground area is small and the decoder’s task reduces to placing a compact, correctly shaped response inside a mostly empty patch. The high Dice values observed here therefore indicate that the decoder is able to produce sharp, well-localized congestion responses even when the per-cell foreground signal is sparse. Taken together, the three grid scales correspond to qualitatively different per-cell reconstruction regimes (cell-filling, boundary-dominant, and blob-inside-cell), and SparseQueryDecoder maintains high Dice in all three.

The qualitative behavior in Figure 9 further supports this interpretation. In the small-grid row, the reconstructed cells fill the patch in close agreement with the ground-truth coverage. In the medium-grid row, the predicted responses are compact and their overall shapes follow the ground-truth blobs, with smoother boundaries than the binary ground truth. Such boundary smoothing is a natural property of a probabilistic decoder operating on hard binary targets, and it fully accounts for the slight gap from the cell-filling regime. In the large-grid row, the model produces small but well-centered blobs whose shapes match the ground truth, with the surrounding background remaining essentially zero. Across all three regimes, the predicted responses consistently concentrate around the true congestion area, with no instances of missed detections or diffuse over-activation. The remaining per-cell discrepancy from unity Dice is therefore attributable to boundary smoothness rather than structural mislocalization, indicating that the decoder reliably captures the correct spatial layout of each congestion region.

This behavior reflects the architectural design of SparseQueryDecoder. Because each high-resolution reconstruction is generated by a learnable query that decodes the local density conditioned on the coarse activation, the decoder is forced to learn a scale-agnostic mapping from coarse evidence to fine-grained spatial structure. The query mechanism allows the same module to operate in the cell-filling regime (where the local response should occupy almost the entire output patch), the boundary-dominant regime (where the response should be a compact blob with sharp edges), and the blob-inside-cell regime (where most of the output patch should remain background). The strong per-cell Dice observed across all three grid scales therefore reflects the complementary strengths of this design: the query-based refinement decouples reconstruction quality from the grid-specific spatial scale, while the conditioning on the coarse activation ensures that the predicted response remains anchored to true density-critical regions.

Overall, the per-cell analysis complements the whole-map results in Table 6: the global accuracy of SparseQueryDecoder is built on individually well-reconstructed cells rather than on averaging effects, and the model is neither biased toward a specific grid configuration nor dependent on a particular foreground-to-background ratio. It thus operates as a grid-robust, scale-flexible refinement module that converts CoarseSTFormer’s coarse density-critical candidates into high-resolution responses with consistently high fidelity, which is precisely the property required of the Stage 2 component in the proposed coarse-to-fine framework.

#### 5.3.3. Comparison with Single-Stage Dense Baselines

To position the proposed coarse-to-fine framework against conventional design choices, we compare it with four single-stage dense baselines that directly predict the high-resolution density map from high-resolution spatio-temporal inputs: SimVP-HR [10], ViT-HR [11], SwinHR [12], and MambaHR [13]. All baselines have the same input format, output resolution, training schedule, and supervision as the proposed model so that the comparison reflects the effect of the architectural choice rather than incidental factors. We report the mean and standard deviation of the high-resolution F1 score over 100 random held-out validation samples per grid scale, with every model evaluated at a single fixed threshold τ=0.5 applied uniformly across all samples and grids. For the proposed framework, each grid is evaluated at its operating cap, M=100 for the small grid and M=50 for the medium and large grids, the same caps used in the cost analysis of Section 5.

As summarized in Table 8, the proposed model attains the highest mean F1 of 0.878, ahead of MambaHR (0.872), SwinHR (0.857), and SimVP-HR (0.856), while ViT-HR trails substantially at 0.407. More telling than the mean itself is how it is achieved. The proposed model is the only method that stays uniformly strong across all three grids, leading the small and medium settings and remaining within a small margin of the best baseline in the large setting. It also carries the smallest variance in every grid, against dense baselines whose spread is consistently larger and, for the weaker models, several times so. Because the three grid scales correspond to qualitatively different per-cell regimes, namely cell-filling, boundary-dominant, and blob-inside-cell as analyzed in the previous per-cell study, this combination of high mean and low variance indicates that the proposed framework adapts to changing reconstruction conditions more reliably than any single dense baseline, each of which peaks only in the regime that happens to suit its architectural prior.

In the small grid (s=5×), the proposed model attains the best F1 of 0.861, well ahead of the dense baselines, which cluster between 0.818 and 0.828. This is the regime in which dense baselines must distribute representational capacity uniformly over the entire 200×200 output grid, even though most of the spatial domain corresponds to background. As discussed in our motivation, such uniform allocation weakens the effective supervision on density-critical pixels, since the loss and gradient signals are dominated by easy background samples. The proposed framework instead restricts Stage 2 computation to candidate regions selected by Stage 1, focusing both representation and supervision on density-critical regions. The proposed model also carries the smallest variance in this regime, indicating that single-stage methods become markedly more sensitive to the spatial distribution of congestion regions as the candidate space grows.

In the medium grid (s=10×), the proposed model and MambaHR are effectively tied at the top (0.876 versus 0.875), with SwinHR close behind. This grid corresponds to the boundary-dominant regime, in which each cell contains a compact blob surrounded by background and precise boundary delineation becomes the dominant difficulty. MambaHR’s structured state-space mixing operates at a fixed resolution and is well matched to this regime. This strength, however, does not carry over to the small grid, where MambaHR falls back to the level of the other dense baselines while the proposed model retains its lead, indicating that its single-resolution prior is less flexible across reconstruction regimes than the query-based refinement in SparseQueryDecoder. SwinHR follows a similar pattern, competitive in the medium grid but degrading in the small grid where its fixed local window cannot fully cover the larger spatial extent of each congestion blob.

In the large grid (s=15×), MambaHR reports the best F1 (0.914), with SimVP-HR close behind and the proposed model within a small margin. This is the blob-inside-cell regime, in which the foreground is sparse and well-separated within a mostly empty patch. Under such conditions, dense prediction effectively reduces to localized hotspot regression, and a model with a strong background prior can suppress most of the output and concentrate its response on the small blob region. This explains why even the relatively simple SimVP-HR becomes competitive here. The finding that the proposed model stays within a small margin of the best dense baseline shows that high-resolution decoding restricted to a compact set of Stage 1 candidates is sufficient to match dense full-grid processing in this regime and, as quantified in the following subsection, at a small fraction of its computational cost.

ViT-HR follows a distinctly different pattern, with F1 collapsing from the small grid to the medium and large grids, accompanied by a steadily growing variance. This indicates a structural rather than a sample-specific failure. It is consistent with the known limitation of plain global self-attention in dense prediction: without an explicit locality prior, such as the depth-wise convolution branch and density-guided local bias in CoarseSTFormer or the hierarchical window structure in SwinHR [12], plain ViT [11] cannot delineate compact congestion regions from a uniform background. The dominance of background tokens during training further biases its attention toward diffuse responses, and this effect worsens as the grid becomes coarser and the foreground grows sparser within each patch. This observation reinforces a central motivation of the proposed framework: locality-aware mechanisms are essential for high-resolution crowd density reconstruction, whether realized through architectural priors or, as in our case, through explicit candidate selection.

**Figure 10:**
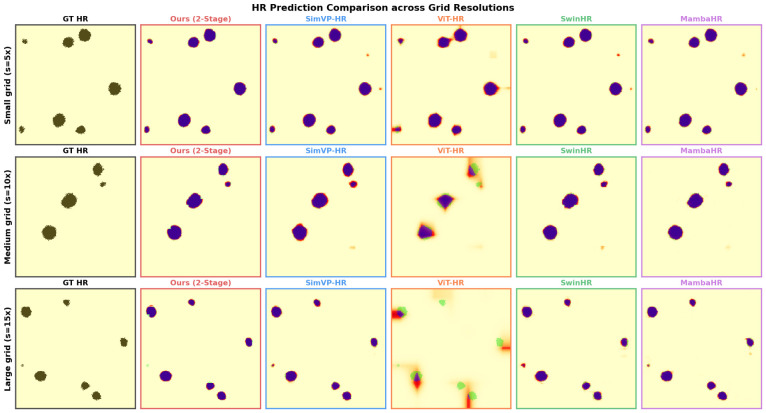
Qualitative comparison of high-resolution density prediction across grid resolutions. From the top row to the bottom row, the results correspond to grid scales s=5×, s=10×, and s=15×, respectively. In each row, the first column shows the high-resolution ground truth, and the remaining columns show the predictions of the proposed two-stage framework, SimVP-HR, ViT-HR, SwinHR, and MambaHR. The color scale encodes the predicted density-critical response, with darker regions indicating higher response.

The qualitative comparison in Figure 10 is consistent with the quantitative trends. In the small grid (top row), most methods recover the congestion blobs at approximately correct locations, but ViT-HR already shows residual halo activations and tends to merge nearby blobs in the upper part of the scene. In the medium grid (middle row), the proposed model, SwinHR, and MambaHR produce compact responses aligned with the ground-truth blobs, whereas ViT-HR generates diffused responses with elongated artifacts that bridge across separate congestion regions. In the large grid (bottom row), the proposed model produces well-centered compact blobs that match the ground truth, while ViT-HR collapses into large, structurally incorrect response regions that occupy a substantial portion of the background. The proposed model is the only method whose qualitative behavior remains visually consistent across the three regimes.

Overall, no single-stage dense baseline performs uniformly well across the three regimes; each peaks only where its architectural prior matches the dominant difficulty of a given grid, as seen in the cross-grid swings of MambaHR (0.828 → 0.875 → 0.914) and SimVP-HR (0.818 → 0.837 → 0.912). The proposed framework instead holds the highest mean F1 and the lowest variance in every grid. It matches the strongest dense baseline in accuracy and, as the next subsection shows, at a small fraction of the computation and energy.

### 5.4. Comparison of Energy, Latency, and Computational Cost

Beyond reconstruction quality, the practical value of the proposed framework rests on its inference cost. Because the two-stage decomposition restricts dense high-resolution computation to the sparse set of active cells identified by CoarseSTFormer, the proposed framework is expected to require substantially less computation than the single-stage dense baselines at deployment, regardless of training. We quantify this along three axes: parameter count, per-sample FLOPs, and wall-time and energy on GPU. For every model under comparison, we run inference on N=100 randomly drawn test samples of the small-grid configuration (s=5×, HR=200×200).

Before reporting absolute cost, we examine how the cap *M* on the number of Stage 2 calls trades accuracy against computation, since *M* both bounds the cost of the refinement stage and determines how many Stage 1 candidates are passed on for reconstruction. Table 9 reports, for each grid scale, the high-resolution $F_{1}$ at τ=0.5, the Stage 1 cell coverage of the ground-truth active set, and the per-sample FLOPs as *M* is varied. Two distinct effects govern the choice of *M*. Cell coverage reaches 100% as soon as *M* exceeds the number of ground-truth active cells, at which point no density-critical cell is dropped before refinement; this is the property that directly rules out cap-induced misses. Reconstruction quality, however, saturates at a somewhat larger *M*, where each retained crop is large enough to cover its congestion region in full. We therefore set the operating cap slightly beyond the coverage point, at the value where $F_{1}$ flattens, so that the cap guards both against missed cells and against truncated reconstructions.

The saturation point depends on how widely the active cells are distributed, which in turn depends on the grid scale. In the small grid, the active set is the most dispersed, averaging roughly seventy cells per scene, so coverage is completed only around M=75 and $F_{1}$ continues to rise until M=100. In the medium and large grids, the active cells are fewer and more concentrated, and both coverage and $F_{1}$ saturate by M=50 or earlier. We therefore adopt a grid-dependent cap, M=100 for the small grid and M=50 for the medium and large grids, rather than a single constant. This is a direct expression of the framework’s sparsity-aware design: the amount of high-resolution computation is matched to how widely congestion is spread over the scene, allocating more refinement where the active region is broad and less where it is compact. The per-sample FLOPs in Table 9 grow with the grid scale at a fixed *M* because a coarser grid assigns a larger physical area to each crop, making every Stage 2 call more expensive even though fewer calls are made.

The three cost axes in Table 10 are internally consistent and collectively favor the proposed framework. On the parameter axis, the proposed two-stage framework uses 2.96 M parameters in total, smaller than three of the four baselines and only slightly above the smallest one (MambaHR, 2.65 M). On the FLOPs axis, the gap is far wider: at its small-grid operating cap of M=100, the proposed framework spends about 8.3 G FLOPs per sample, whereas the four dense baselines require 31 to 442 G, roughly 3.8 to 54× more arithmetic. This follows directly from the architectural design, as the dense baselines apply their full forward pass over the entire 200×200 domain, while the proposed framework operates on a 40×40 low-resolution input at Stage 1 and on small crops at Stage 2, with the latter invoked only on active cells.

**Figure 11:**
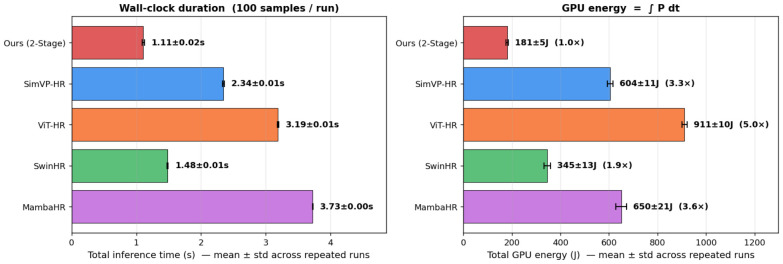
Aggregate inference cost on the small-grid configuration, averaged over five 100-sample runs. Left: total wall-clock duration per model (mean ± std). Right: total GPU energy per model with the ratio to the proposed framework reported next to each bar. Standard deviations remain below a few percent of the mean for every model.

The measured wall-time and energy, shown in Figure 11, agree with the FLOPs picture. The proposed framework completes the 100-sample inference in 1.11±0.02 s while consuming 181±5 J, which is 1.3 to 3.4× faster and 1.9 to 5.0× more energy-efficient than the dense baselines. The across-run standard deviation stays within a few percent of the mean for every model, indicating that the differences reflect architectural cost rather than measurement noise. The largest energy gap is against ViT-HR, which pairs a high sustained power draw with a long inference duration. The proposed framework also draws the lowest mean GPU power, never reaching the saturated ≈310 W regime that the dominant baselines settle into, consistent with it never sustaining a dense high-resolution compute load at any point in its forward pass.

Taken together, the proposed framework holds a consistent cost advantage across parameters, FLOPs, wall-time, and energy. Considering the reconstruction-quality results of the preceding subsection, where it matches the strongest dense baseline on average at the same operating cap used here, this shows that the two-stage decomposition does not trade quality for efficiency. It preserves quality while moving computation off the dense high-resolution canvas and onto the compact set of density-critical regions identified by CoarseSTFormer.

## 6. Conclusions

This paper presented a two-stage coarse-to-fine framework for crowd density prediction that explicitly exploits the spatio-temporal sparsity of congestion events. The framework couples CoarseSTFormer, which performs efficient global screening at low resolution to identify density-critical candidate regions, with SparseQueryDecoder, which performs sparse high-resolution reconstruction only on the selected candidates. By decoupling global screening from local refinement, the framework concentrates both computation and supervision on safety-relevant regions of the BEV grid.

Simulation experiments confirmed that this design yields reliable localization and reconstruction across the evaluated grid configurations while substantially reducing inference cost compared with single-stage dense baselines. The framework thus provides a practical predictive module for digital twin-based crowd safety monitoring.

Future work will extend the evaluation to real-world crowd video benchmarks, incorporate uncertainty quantification to express confidence over density-critical regions, and integrate the framework into downstream tasks such as long-horizon crowd flow forecasting and anomaly-aware safety monitoring.

## Figures and Tables

**Table 1:** Comparison of AI-based crowd management approaches.

Method Category	Future	Environment	Sparsity
	Prediction	Level	Aware
Crowd counting/density estimation [2,3,19]	×	△	×
Anomaly/behavior detection [20,21]	×	△	×
Pedestrian trajectory prediction [23,24,25]	✓	×	×
General spatio-temporal predictors [10,11,12,13]	✓	✓	×
Ours (Two-stage coarse-to-fine)	✓	✓	✓

**Table 2:** Aggregate Stage 1 localization performance of CoarseSTFormer across the 646 active validation scenes, grouped into five density bins by GT active-cell count and labeled #10000 through #20000 to denote increasing congestion. Precision, recall, and $F_{1}$ are reported as mean ± std at a single fixed threshold τ=0.5. Each $F_{1}$ entry is the mean of per-scene $F_{1}$ values rather than the harmonic mean of the column-averaged precision and recall.

Case	GT-Cell Bin	*n*	$\bar{\mathrm{GT}}$	$\bar{\mathrm{Det}}$	Precision	Recall	$F_{1}$
#10000	1–4	155	2.2	2.3	0.879±0.256	0.897±0.248	0.874±0.239
#12000	5–12	156	8.1	8.4	0.943±0.087	0.958±0.080	0.946±0.067
#14000	13–28	135	19.3	19.7	0.935±0.058	0.954±0.051	0.943±0.042
#16000	29–55	154	40.7	41.7	0.946±0.036	0.967±0.031	0.956±0.026
#20000	56+	46	61.8	62.8	0.951±0.030	0.967±0.022	0.959±0.023

**Table 3:** Representative coarse-stage localization performance of CoarseSTFormer across different crowd densities. Each row corresponds to a scene whose $F_{1}$ falls near the bin mean, with all entries computed at τ=0.5. The explicit true-positive (TP) column makes the (GT, Det, TP) triple consistent with the reported precision, recall, and $F_{1}$.

Case	GT Cells	Detected Cells	TP	Precision	Recall	F1
#10000	3	4	3	0.750	1.000	0.857
#12000	11	12	11	0.917	1.000	0.957
#14000	28	29	27	0.931	0.964	0.947
#16000	46	50	46	0.920	1.000	0.958
#20000	73	75	71	0.947	0.973	0.959

**Table 4:** Representative Stage 1 localization performance under different prediction horizons.

Prediction Horizon	GT Cells	Detected Cells	Precision	Recall	F1
2	75	77	0.935	0.960	0.947
5	101	103	0.951	0.970	0.961
10	75	84	0.798	0.893	0.843

**Table 5:** Representative Stage 1 localization performance under different grid resolutions.

Grid Size	GT Cells	Detected Cells	Precision	Recall	F1
5	43	46	0.891	0.953	0.921
10	13	14	0.929	1.000	0.963
15	18	19	0.947	1.000	0.973

**Table 6:** Whole-map high-resolution reconstruction performance of SparseQueryDecoder under different grid resolutions.

Grid Scale	LR Size	HR Size	Positive/Active Cells	Dice	IoU
s=5×	40×40	200×200	76/124	0.926	0.862
s=10×	20×20	200×200	16/43	0.891	0.804
s=15×	14×14	210×210	6 /23	0.897	0.813

**Table 7:** Per-cell high-resolution reconstruction Dice for representative active cells under different grid resolutions.

Grid Scale	Per-Cell Patch	Per-Cell Dice (4 Representative Cells)	Mean
s=5×	15×15	0.961/0.967/0.955/0.958	0.960
s=10×	30×30	0.893/0.919/0.895/0.926	0.908
s=15×	45×45	0.953/0.906/0.954/0.952	0.941

**Table 8:** Mean F1 (±standard deviation) of high-resolution density prediction across 100 random held-out validation samples per grid, with all models evaluated at a single fixed threshold τ=0.5. The proposed framework is evaluated at its per-grid operating cap. The best score in each grid is marked in bold.

Model	Small (s=5×)	Medium (s=10×)	Large (s=15×)	Mean
**Ours (2-Stage)**	0.861±0.047	0.876±0.028	0.896±0.032	0.878
SimVP-HR	0.818±0.086	0.837±0.039	0.912±0.017	0.856
ViT-HR	0.689±0.134	0.263±0.176	0.268±0.252	0.407
SwinHR	0.827±0.073	0.861±0.036	0.882±0.027	0.857
MambaHR	0.828±0.086	0.875±0.028	0.914±0.012	0.872

**Table 9:** Effect of the Stage 2 cap *M* across grid scales. For each grid, the high-resolution $F_{1}$ at τ=0.5, the Stage 1 cell coverage of the ground-truth active set, and the per-sample FLOPs are reported as *M* is varied. Cell coverage reaching 100% indicates that no ground-truth active cell is dropped, while $F_{1}$ saturates at a slightly larger *M* where the retained crops fully cover each congestion region. The operating cap selected for each grid is marked in bold. In the large grid, M=30 and M=50 give identical results because Stage 1 yields only about 18 candidates per scene on average, leaving the cap non-binding once coverage is complete.

Grid	*M*	$F_{1}$ (μ±σ)	Cell Coverage (%)	FLOPs (G)
Small (s=5×)	30	0.729±0.119	84.1	4.08
	50	0.808±0.070	97.2	5.61
	75	0.846±0.050	100.0	7.15
	100	0.861±0.047	100.0	8.26
	150	0.867±0.047	100.0	9.12
Medium (s=10×)	20	0.795±0.082	100.0	8.70
	30	0.846±0.051	100.0	11.72
	50	0.876±0.028	100.0	14.92
	75	0.876±0.028	100.0	15.05
	100	0.876±0.028	100.0	15.05
Large (s=15×)	15	0.874±0.053	100.0	15.64
	20	0.891±0.037	100.0	18.26
	30	0.896±0.032	100.0	19.29
	50	0.896±0.032	100.0	19.29

**Table 10:** Per-architecture inference cost on the small-grid configuration. Parameter counts and FLOPs are computed analytically. Wall-clock duration and GPU energy are measured on a single L40S GPU over five independent 100-sample inference runs, reported as mean ± std. For the proposed framework, the per-sample FLOPs entry corresponds to Stage 1 plus M=100 Stage 2 calls, the operating cap for the small grid. The rightmost column reports the energy ratio relative to the proposed framework. Bold values denote the proposed framework.

Model	Params	FLOPs	Duration (μ±σ)	Energy (μ±σ)	Ratio
**Ours (2-Stage)**	2.96 M	8.26 G	1.11±0.02 s	181±5 J	1.0×
SwinHR	4.38 M	39.9 G	1.48±0.01 s	345±13 J	1.9×
SimVP-HR	6.17 M	442.4 G	2.34±0.01 s	604±11 J	3.3×
MambaHR	2.65 M	41.6 G	3.73±0.00 s	650±21 J	3.6×
ViT-HR	7.23 M	31.1 G	3.19±0.01 s	911±10 J	5.0×

## Data Availability

The results presented in this study are provided on a limited basis upon request of the corresponding author. Data may not be made publicly available for reasons of data protection by the relevant organizations.
